# Antibody recognition of complement factor H reveals a flexible loop involved in atypical hemolytic uremic syndrome pathogenesis

**DOI:** 10.1016/j.jbc.2022.101962

**Published:** 2022-04-20

**Authors:** Takanori Yokoo, Aki Tanabe, Yoko Yoshida, Jose M.M. Caaveiro, Makoto Nakakido, Yoichiro Ikeda, Yoshihiro Fujimura, Masaneori Matsumoto, Kevin Entzminger, Toshiaki Maruyama, C.J. Okumura, Masaomi Nangaku, Kouhei Tsumoto

**Affiliations:** 1 Department of Chemistry and Biotechnology, School of Engineering, The University of Tokyo, Bunkyo-ku, Tokyo, Japan; 2 Department of Bioengineering, School of Engineering, The University of Tokyo, Bunkyo-ku, Tokyo, Japan; 3 Division of Nephrology and Endocrinology, The University of Tokyo, Bunkyo-ku, Tokyo, Japan; 4 Department of Global Healthcare, Graduate School of Pharmaceutical Sciences, Kyushu University, Higashi-ku, Fukuoka, Japan; 5 Department of Blood Transfusion Medicine, Nara Medical University, Kashihara, Nara, Japan; 6 Abwiz Bio Inc, San Diego, California, USA; 7 The institute of Medical Science, The University of Tokyo, Minato-ku, Tokyo, Japan

**Keywords:** aHUS, CFH, autoantibody, complement system, autoimmune disease, VHH, MD simulation, X-ray crystallography, isothermal titration calorimetry, surface plasmon resonance, AAbs, autoantibodies, aHUS, atypical hemolytic uremic syndrome, AP, alternative pathway, C3, complement component 3, CCP, complement control protein, CD, circular dichroism, CDR, complementarity-determining region, CFH, complement factor H, CFI, complement factor I, iC3b, inactive C3b, IMAC, immobilized metal affinity chromatography, ITC, isothermal titration calorimetry, MD, molecular dynamics, RMSF, root mean square fluctuation, SA, sialic acid, SEC, size exclusion chromatography, SPR, surface plasmon resonance, SRBC, sheep red blood cell

## Abstract

Atypical hemolytic uremic syndrome (aHUS) is a disease associated with dysregulation of the immune complement system, especially of the alternative pathway (AP). Complement factor H (CFH), consisting of 20 domains called complement control protein (CCP1-20), downregulates the AP as a cofactor for mediating C3 inactivation by complement factor I. However, anomalies related to CFH are known to cause excessive complement activation and cytotoxicity. In aHUS, mutations and the presence of anti-CFH autoantibodies (AAbs) have been reported as plausible causes of CFH dysfunction, and it is known that CFH-related aHUS carries a high probability of end-stage renal disease. Elucidating the detailed functions of CFH at the molecular level will help to understand aHUS pathogenesis. Herein, we used biophysical data to reveal that a heavy-chain antibody fragment, termed VHH4, recognized CFH with high affinity. Hemolytic assays also indicated that VHH4 disrupted the protective function of CFH on sheep erythrocytes. Furthermore, X-ray crystallography revealed that VHH4 recognized the Leu1181–Leu1189_CCP20_ loop, a known anti-CFH AAbs epitope. We next analyzed the dynamics of the C-terminal region of CFH and showed that the epitopes recognized by anti-CFH AAbs and VHH4 were the most flexible regions in CCP18-20. Finally, we conducted mutation analyses to elucidate the mechanism of VHH4 recognition of CFH and revealed that VHH4 inserts the Trp1183_CCP20_ residue of CFH into the pocket formed by the complementary determining region 3 loop. These results suggested that anti-CFH AAbs may adopt a similar molecular mechanism to recognize the flexible loop of Leu1181-Leu1189_CCP20_, leading to aHUS pathogenesis.

Atypical hemolytic uremic syndrome (aHUS) is a disease characterized by microangiopathic hemolytic anemia, thrombocytopenia, and acute kidney injury (1). aHUS is associated with dysregulation of the complement system caused by genetic or acquired defects. Selective activation of the alternative pathway is involved in pathogenesis (1, 2). In the alternative pathway, deposition of the complement protein C3b leads to activation of the complement cascade that subsequently may initiate the formation of the membrane-attack complex (1, 3). On host cells, activation of the alternative pathway is controlled by complement factor H (CFH) and complement factor I (CFI). CFH is a cofactor for the protease CFI that degrades C3b, resulting in inactive C3b (iC3b). CFH also accelerates the irreversible decay of C3bBb (an enzymatic complex that cleaves C3 to generate more C3b in a positive-feedback loop) into C3b and Bb (1, 4). aHUS is caused by overactivation of the alternative pathway due to the dysfunction of complemental proteins including C3b, CFH, and CFI, among other complement proteins (1, 2). Although CFH, CFI, C3, and other complement-related factors have been reported pathogenic (5, 6), CFH has the strongest impact on the pathogenesis of aHUS because CFH-associated aHUS carries a high probability of loss of renal function or end-stage renal disease (70–80%) (1).

CFH (155 kDa) forms a linear, chain-like structure consisting of twenty domains called complement control protein (CCP) 1 to 20 (CCP1-20), each comprising ∼60 residues (7, 8). Two regions of CFH bind to C3b: the first four domains (CCP1-4) and the last two domains (CCP19–20) (9). In particular, CCP19-20 binds to C3d, which is part of C3b (9). Moreover, CFH recognizes glycosaminoglycans and sialic acid (SA) glycans as self-markers (10). Among these two glycans, SA is recognized by CFH using domains CCP7 and CCP20 (11, 12). The crystal structure of SA with CCP19-20 and C3d was determined (10). Presence and absence of SA binding have been thought to be important for controlling an alternative pathway by CFH and self-recognition of the complementary system (3, 10, 13).

In aHUS derived from CFH abnormality, CFH mutations (1, 2) and development of anti-CFH autoantibodies (AAbs) (1, 14, 15) have been reported (Fig. 1). CFH mutations in patients with aHUS have been often observed in the C-terminal, a region that comprises domains CCP19-20 (1, 5, 6). Interestingly, this very same region is a major autoantibody-binding site (16, 17, 18, 19, 20). The relationship between CFH mutations and aHUS has been analyzed and elucidated using site-directed mutagenesis focusing on the cofactor active site based on crystal structures of CFH in the unliganded form or in complex with C3b or SA (7, 9, 10, 21). Moreover, some reports have suggested that the development of anti-CFH AAbs is associated with the deletion of the CFH-related protein 1, whose amino acid sequence at the C-terminus three domains, CCP3-5 of CFHR1, is almost identical to that of CCP18-20 of CFH (16, 18, 19, 22, 23, 24). Furthermore, based on the analyses of the hemolytic effect using sheep erythrocytes, several studies have reported that anti-CFH AAbs recognizing CCP19-20 inhibited CFH functions and showed hemolytic activity (16, 17, 19, 20, 25). Among the studies characterizing anti-CFH AAbs, two reports have identified the epitopes of anti-CFH AAbs at the molecular level, one using peptide fragments of CFH (26) and the other using CFH mutants (27). Regions Arg1182-Leu1189 and Arg1210-Arg1215 in CCP20 have been identified as the epitopes in both studies. On the other hand, the recognition mechanism, function, and production mechanism of anti-CFH AAbs remain to be elucidated.

**Figure 1:**
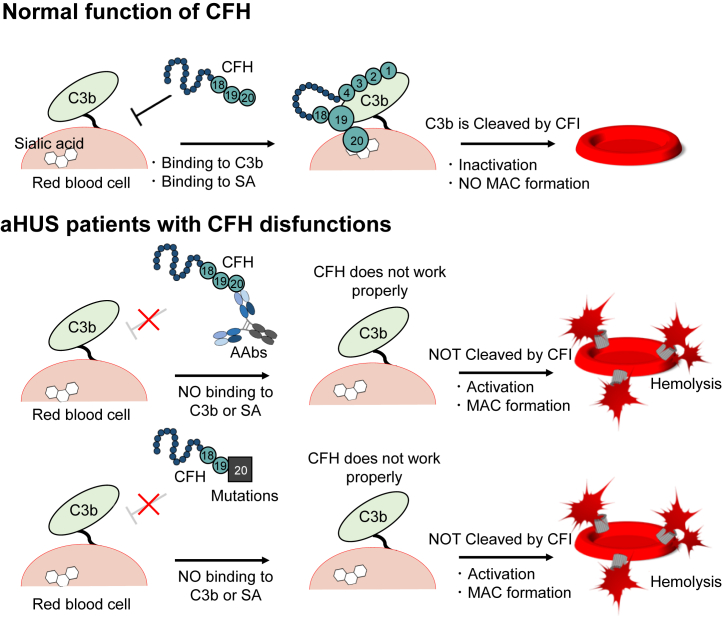
**Schematic view of function of CFH (top panel) and CFH dysfunction seen in patients with aHUS (center and bottom panels).** CFH plays a role in regulating complement activation on *red* blood cells. In patients with aHUS, autoantibodies against CFH (*middle panel*) or mutations of CFH (*bottom panel*) have been reported as CFH dysfunctions. aHUS, atypical hemolytic uremic syndrome; CFH, complement factor H.

Each CCP domain adopts a *β*-sandwich-type fold including four consensus cysteine residues forming two disulfide bonds and having an overall prolate spheroidal shape with N- and C- terminal lying close to opposite poles of the long axis (8, 28). According to previous investigations, CFH does not display the typical circular dichroism (CD) spectrum characteristic of *α*-helices and *β*-sheets (29). Therefore, it is suggested that CFH may display a flexible structure in solution based on the features of the crystal structure (7, 30, 31) and the CD spectra (29). Nonetheless, the detailed dynamic behavior of the CCP domains of CFH in solution is still unknown.

In this study, we generated a VHH antibody that binds tightly to the highly flexible Leu1181–Leu1189 loop of CCP20, which is the region autoantibodies often recognize in antibody-mediated aHUS. The hemolytic analysis showed the ability of the VHH4 to inhibit the binding of SA to CFH. To characterize the molecular basis of the interaction between this antibody (termed VHH4) and the antigen, we employed X-ray crystallography and other biophysical techniques. Based on our data, we discuss the possibility that AAbs in aHUS adopt similar mechanisms to recognize the CFH, leading to pathogenesis.

## Results

### Acquisition of a novel VHH antibody against the C-terminal region of CFH

**Figure 2:**
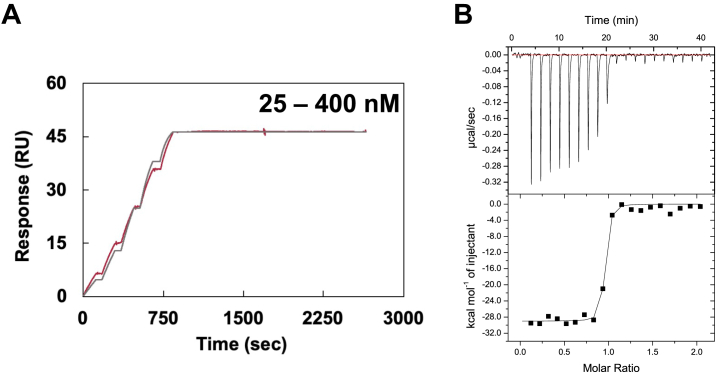
**High-affinity interaction between CFH18-20 and VHH4.***A*, SPR sensorgram. SPR measurements were performed in PBS at pH 7.4 and Tween 20 (0.005%) at 25 °C. CFH was immobilized on a CM5 sensor chip, and VHH4 was flowed as the analyte. Runs were performed using single kinetics method. The *red* and *black lines* correspond to raw data and fitting curves, respectively. *B*, titration of CFH18-20 with VHH4. ITC measurements were conducted in PBS pH 7.4 at 25 °C. Each titration consisted in 20 injections of VHH4 into the cell containing CFH18-20. Each measurement was performed three times independently. CFH, complement factor H; ITC, isothermal titration calorimetry; SPR, surface plasmon resonance.

To elucidate how anti-CFH AAbs are associated with aHUS, we tried to obtain novel antibodies that recognize the C-terminal region of CFH. We prepared recombinant protein corresponding to domains CFH18-20 (Figs. S1 and S2) and immunized a specimen of alpaca. After confirmation of serum response by ELISA, we isolated B cells from blood, RNAs were extracted, and a VHH phagemid library was constructed. We successfully obtained ten VHH clones by several rounds of biopanning relying on phage display. Six of the ten VHHs were successfully expressed in *E.coli* and purified by immobilized metal affinity chromatography (IMAC) and size exclusion chromatography (SEC) (Fig. S3). The purified VHHs were subjected to surface plasmon resonance (SPR) to verify which clones were able to recognize CFH (Fig. S4). CFH18-20 was immobilized on the sensor chip, and VHHs were injected as the analyte. Among the VHHs examined, VHH4 interacted with CFH18-20 with remarkably high affinity (*K*_D SPR_ = 1.71 ± 0.66 pM) and a rather slow dissociation rate constant (Figs. 2, S4 and Table 1). The interaction was further characterized by isothermal titration calorimetry (ITC) (Fig. 2 and Table 2). Although the *K*_D_ value could not be accurately determined due to technical limitations under the conditions of ultrahigh affinity, the result indicated that the interaction was exothermic, suggesting high specificity of VHH4.

**Table 1:** Kinetic parameters of VHH4 and mutants evaluated by SPR^a^

Mutation site	*k*_on_ (10^3^/Ms)^b^	*k*_off_ (10^−5^/s)^b^	*K*_D (SPR)_ (nM)^b^
WT	31.4 ± 1.60	0.00553 ± 0.00223^c^	0.00171 ± 0.00066^c^
Trp1183A_CCP20_	N.D.^d^	N.D.	N.D.
Tyr95A_VHH4_	30.4 ± 4.00	0.442 ± 0.226^c^	0.166 ± 0.083^c^
Pro102A_VHH4_	23.7 ± 2.80	0.407 ± 0.397^c^	0.219 ± 0.215^c^
Leu104A_VHH4_	11.2 ± 0.70	48.9 ± 0.30	44.1 ± 2.3
Thr108A_VHH4_	17.9 ± 1.20	0.257 ± 0.251^c^	0.146 ± 0.143^c^
Pro110A_VHH4_	9.21 ± 3.60	0.711 ± 0.710^c^	0.504 ± 0.503^c^
Tyr117A_VHH4_	11.3 ± 1.80	0.0539 ± 0.0516^c^	0.498 ± 0.496^c^
Tyr118A_VHH4_	15.8 ± 3.80	2980 ± 465	2200 ± 732
Gly119A_VHH4_	62.8 ± 10.2	61.1 ± 18.7	9.18 ± 1.85
Asp121A_VHH4_	4.34 ± 0.40	54.5 ± 11.8	127 ± 30
Trp123A_VHH4_	31.2 ± 4.90	15.9 ± 6.50	5.05 ± 1.89

a All data are the average of three independent SPR and ITC experiments, respectively.

b *k*_on_, *k*_off_ and *K*_D (SPR)_ were determined by SPR.

c Parameters could not be determined with sufficient degree of confidence because of the unusual slow dissociation phase.

d Parameters were not determined.

**Table 2:** Thermodynamic parameters of VHH4 and mutants evaluated by ITC^a^

Mutation site	*N* (Sites)^b^	*K*_D (ITC)_ (nM)^b^	*ΔG* (kcal/mol)^b^	*ΔH* (kcal/mol)^b^	−*TΔS* (kcal/mol)^b^
WT	0.895 ± 0.019	2.01 ± 0.34^c^	−11.89 ± 0.12^c^	−28.85 ± 0.51	16.96 ± 0.62^c^
Trp1183A_CCP20_	N.D.^d^	N.D.	N.D.	N.D.	N.D.
Tyr95A_VHH4_	0.842 ± 0.039	2.27 ± 0.84^c^	−11.90 ± 0.26^c^	−28.18 ± 0.97	16.28 ± 1.00^c^
Pro102A_VHH4_	0.961 ± 0.030	4.11 ± 2.26^c^	−11.63 ± 0.32^c^	−29.61 ± 0.74	17.98 ± 0.63^c^
Leu104A_VHH4_	0.947 ± 0.061	166 ± 40.3	−9.29 ± 0.15	−24.49 ± 0.99	15.21 ± 1.11
Thr108A_VHH4_	0.924 ± 0.089	8.79 ± 3.06^c^	−11.11 ± 0.26^c^	−27.20 ± 0.55	16.09 ± 0.59^c^
Pro110A_VHH4_	0.838 ± 0.032	5.03 ± 0.23^c^	−11.33 ± 0.03^c^	−29.79 ± 2.16	18.46 ± 2.18^c^
Tyr117A_VHH4_	0.933 ± 0.017	5.87 ± 1.15^c^	−11.27 ± 0.27^c^	−28.05 ± 0.52	16.78 ± 0.60^c^
Tyr118A_VHH4_	0.947 ± 0.041	410 ± 26.4	−8.72 ± 0.04	−19.42 ± 0.33	10.70 ± 0.36
Gly119A_VHH4_	0.865 ± 0.026	7.26 ± 3.27^c^	−11.23 ± 0.25^c^	−19.40 ± 0.61	8.17 ± 0.78^c^
Asp121A_VHH4_	1.002 ± 0.019	361 ± 64.9	−8.83 ± 0.10	−29.46 ± 1.14	20.63 ± 1.23
Trp123A_VHH4_	0.866 ± 0.036	3.92 ± 0.93^c^	−11.51 ± 0.15^c^	−20.95 ± 0.63	9.45 ± 0.48^c^

a All data are the average of three independent ITC experiments, respectively.

b Binding ratio (*N*), affinity (*K*_D(ITC)_), Gibbs free energy changes (*ΔG*), enthalpy changes (*ΔH*), and entropy changes (−*TΔS*) were determined by ITC.

c Parameters could not be determined with sufficient degree of confidence because of the high affinity.

d Parameters were not determined.

### VHH4 induced hemolysis by the inhibition of CFH binding to SA on the cell surface

We evaluated the ability of VHH4 to inhibit CFH by a hemolytic assay using nonsensitized sheep red blood cells (SRBCs) that are generally employed to assess the cell-protective function of CFH. The C-terminal region of CFH (CCP19–20) binds to the abundant SA moieties present on the surface of SRBCs and protects erythrocytes from complement-mediated lysis. Thus, the dysfunction of the C-terminal region of CFH causes hemolysis of SRBCs (32).

**Figure 3:**
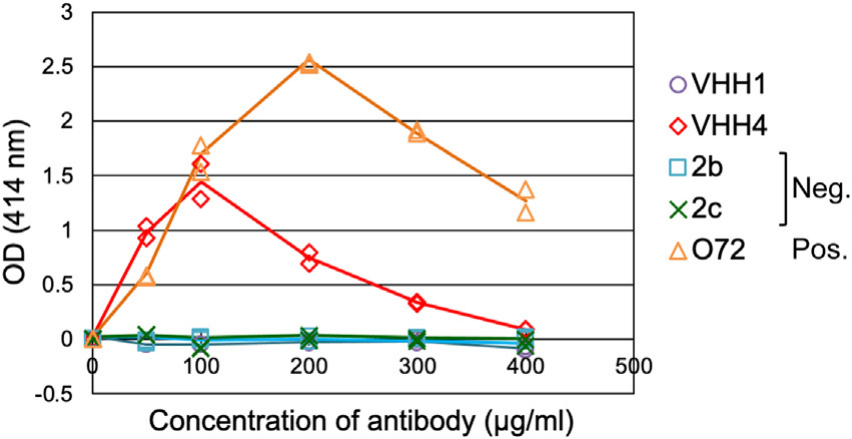
**VHH4 inhibited CFH function and induced hemolysis.** Results of hemolytic assay. Hemolysis was evaluated from the absorbance at 414 nm derived from hemoglobin. O72, a mouse anti-human CFH monoclonal antibody that recognizes CCP18 in the C-terminus of CFH, was employed. This antibody inhibits the adhesion of CFH to cell surfaces and induces enhanced hemolysis and was used as a positive control. 2b and 2c were negative control. VHH1 is an antibody recognizing regions different from that recognized by VHH4. 2b and 2c were negative control. VHH1 is an antibody recognizing regions different from that recognized by VHH4. 2b and 2c are alpaca anti-Lysozyme heavy chain heavy displaying a long hinge (2b) or a short hinge (2c), respectively. Experiment was performed in duplicate. Measured values for each experiment are shown as *dots*, and average values are shown as *line graphs*. CCP, complement control protein; CFH, complement factor H.

As shown in Figure 3, the addition of VHH4 induced hemolysis of SRBCs. We further analyzed the fluid-phase cofactor activity of CFH for CFI-mediated C3b inactivation. As shown in Fig, S5A, the complement C3 is cleaved and inactivated to eventually C3c via C3b and iC3b by cofactor CFH and protease CFI (9). We analyzed the number of fragments derived from C3 cleavage reaction in the presence or absence of VHH4 by SDS-PAGE. The result showed that VHH4 did not inhibit the C3 degradation reaction caused by CFH and CFI. (Fig. S5*B*), implying that VHH4 might have little influence on the CFH binding to C3b or CFI. Added to the fact that first, binding of CFH to both C3b and SA is required for CFH to be fully effective as a complement suppression cofactor to protect erythrocytes from hemolysis and, second, that mutants of CFH at the SA-binding region resulted in significantly weaker protecting activity than that offered by wildtype CFH (11), these results suggested that VHH4 caused hemolysis by inhibiting CFH binding to SA. Interaction analysis between C3d and the complex of CFH18-20 and VHH4 using SPR also showed that CFH still have the binding ability to C3d in forming a complex with VHH4 (Fig. S6).

### Cocrystal structure of the complex with VHH4 reveals interaction through CCP20

**Figure 4:**
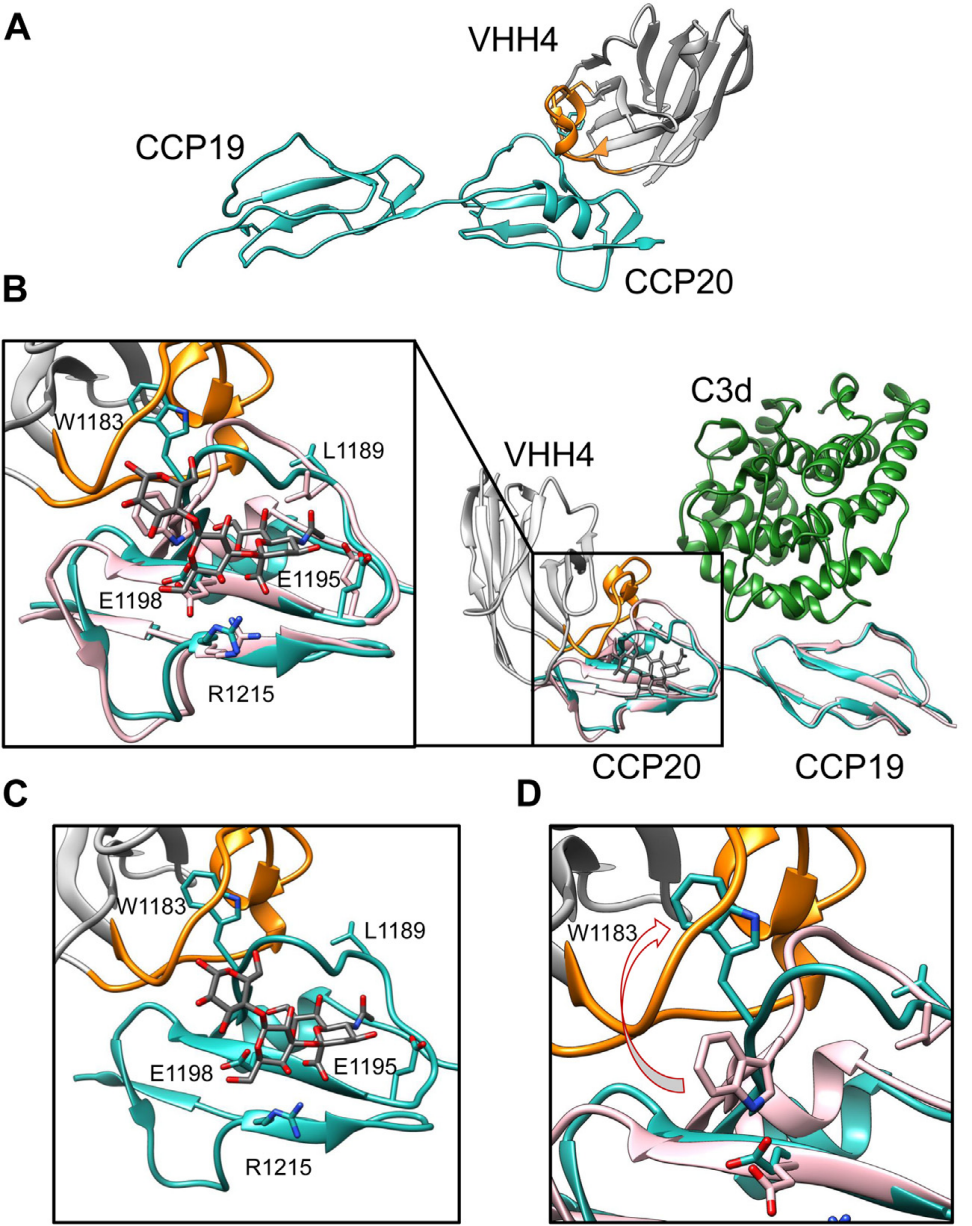
**Crystal structure of CFH18-20 in complex with VHH4.***A*, overview of the structure. The CDR3 of VHH4 is depicted in *orange*. VHH4 and CFH are shown in *gray* and *blue*, respectively. *B*, superposition of VHH4-CFH complex and CFH-C3d-sialic acid (SA) complex (PDB: 4ONT). CFH19-20 bound to VHH4, CFH19-20 bound to C3d and SA, C3d, SA, VHH4 CDR3, and the other regions of VHH4 are colored *pink* and *light blue*, *green*, *dark gray*, *orange*, and *white gray*, respectively. The *left panel* corresponds to the closeup view of the binding interface, highlighting the loop Leu1181–Leu1189 in domain CCP20 of CFH. Domain CCP18 was not observed in our crystal structure. *C*, superposition of VHH4, SA, and CCP20 bound to VHH4. *D*, enlarged figure around Trp1183_CCP20._ The CFH bound to C3d and SA, CFH bound to VHH4, and VHH4 were superposed. CCP, complement control protein; CFH, complement factor H.

**Figure 5:**
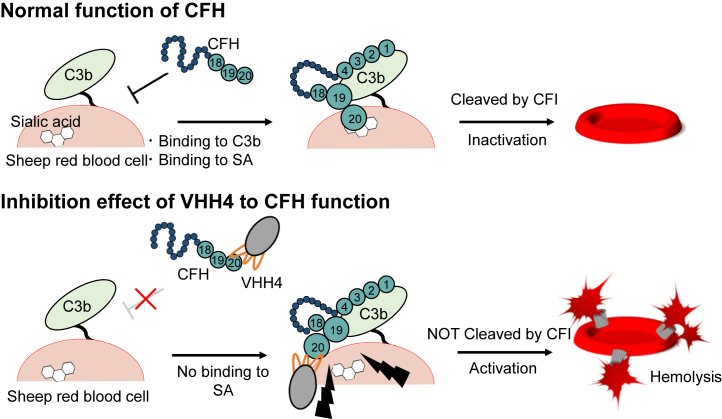
**Inhibition mechanism of VHH4.** Schematic diagram illustrating the function of CCP18-20 (*top panel*) and inhibition mechanism by VHH4 (*bottom panel*). CCP19-20 recognize C3b, whereas CCP20 may also bind to SA on the surface of *red* blood cell. CCP, complement control protein.

To investigate the molecular mechanism by which VHH4 induces hemolysis, we crystallized the complex of the C-terminal region of CFH and VHH4 and determined the structure at a resolution of 2.6 Å (Fig. 4*A*). The crystal structure revealed that VHH4 recognized the loop Leu1181–Leu1189_CCP20_ belonging to the CCP20 domain using complementarity-determining region 3 (CDR3) (Fig. 4*A*). Importantly, this loop corresponds to a region previously reported to be the epitope of anti-CFH AAbs (26, 27). We superimposed the crystal structures of the complex of CFH19-20-VHH4 with the previously reported structure of CFH19-20-C3d-SA complex (PDB: 4ONT) (10). The superimposed structure suggested a steric hindrance between the N-terminal side of CDR3 of VHH4 and SA (Fig. 4, *B* and *C*). Specifically, the binding of VHH4 pushed the loop of CFH toward the side of SA, thereby causing steric hindrance between the loop and SA (Fig. 4, *B* and *C*). In addition, the conformation of the Leu1181–Leu1189_CCP20_ loop was altered by VHH4. The residue Trp1183_CCP20_ belonging to this loop, and necessary for the interaction with SA, is now surrounded by the VHH4 CDR3 loop (Fig. 4, *B* and *D*). Collectively, these results suggested that VHH4 inhibits CFH binding to SA on the surface of the erythrocyte membrane (Fig. 5).

### Molecular dynamics simulations suggest that the two most flexible regions in CFH18-20 correspond to epitopes of AAbs and VHH4

**Figure 6:**
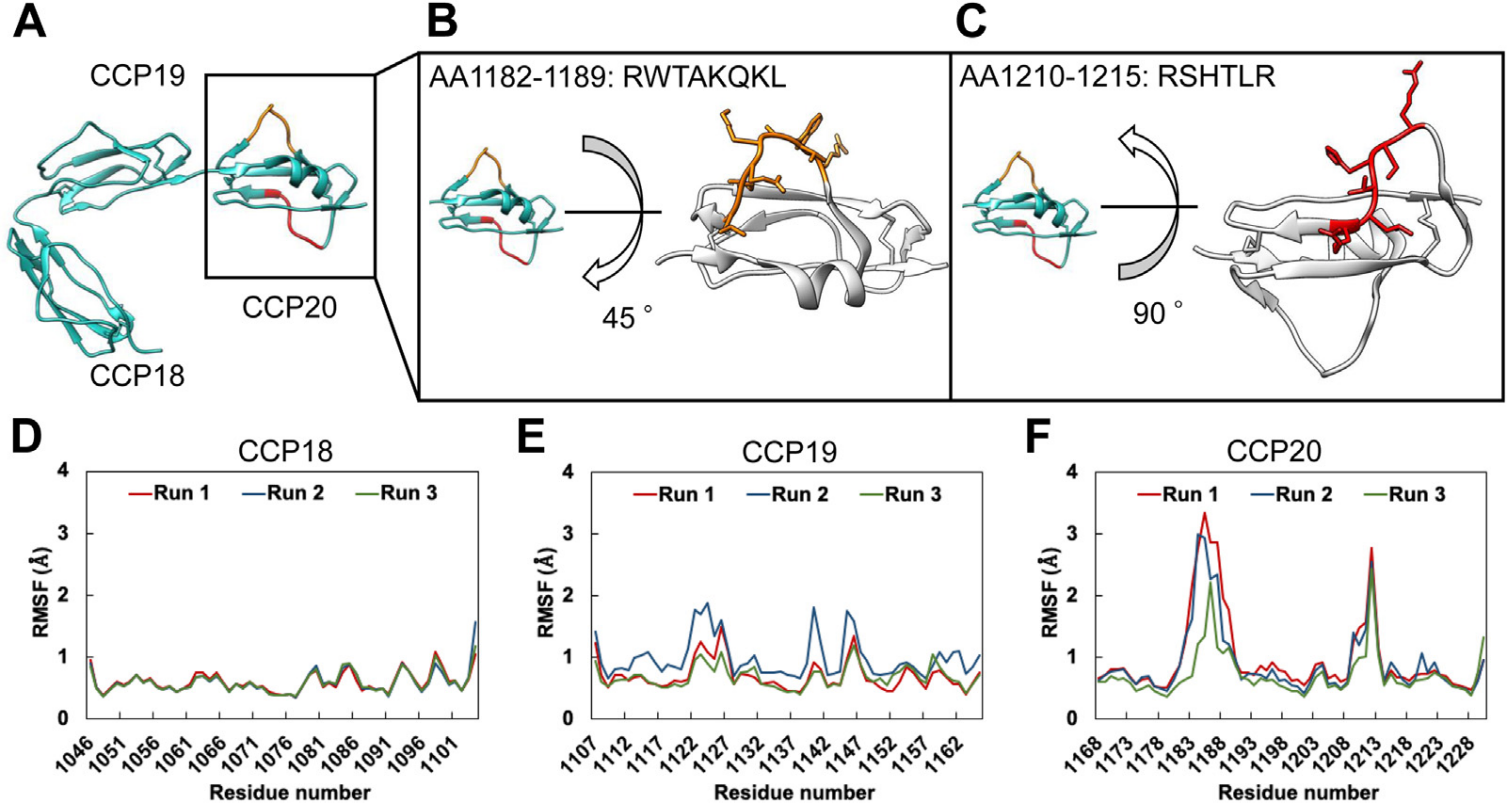
**The epitopes of anti-CFH autoantibodies comprise flexible region in CFH18-20.***A*, crystal structure of CFH CCP18-20 (PDB ID: 3SW0). Two epitopes of previously reported AAbs are shown in *orange* (R1182-L1189) and *red* (R1210-R1215). *B and C*, the position in CCP20 and side chain orientation of Arg1182-Leu1189 and Arg1210-Arg1215. *D*-*F*, RMSF values of each residue in each domain. CCP18, CCP19, and CCP20 comprise residues of 1046 to 1104, 1107 to 1165, and 1168 to 1230 of CFH, respectively. CCP, complement control protein; CFH, complement factor H.

To characterize the L1181–L1189_CCP20_ loop, which is the epitope for VHH4 as well as for AAbs in aHUS, we conducted molecular dynamics (MD) simulations. We performed MD simulations using the crystal structure of CFH18-20 in the unbound form (PDB: 3SW0) (30) (Fig. 6*A*). First, we calculated the RMSD and root mean square fluctuation (RMSF) of the C*α* atoms in each domain (Figs. 6*D* and S7, *B*–*D*). The RMSD and RMSF values revealed that CCP18 was the most rigid domain among these three domains (Figs. 6*D* and S7, *B*–*D*). Intriguingly, large RMSF values were observed, especially in loops Leu1181–Leu1189_CCP20_ and Arg1210-Thr1217_CCP20_ (Fig. 6*D*). It is thus suggested that the Leu1181–Leu1189_CCP20_ loop, which is one of the functional regions, of CFH was also the most flexible regions in the C-terminal region of CFH. The pathogenesis of autoantibody-mediated aHUS is likely to be caused by the binding of AAbs to a functional site of CFH such as this loop, thus inhibiting the CFH binding to the SA moiety. Also, looking into the steps necessary for the generation of AAbs and given that antibodies have the tendency to recognize a rigid portion of antigens (33, 34), this result may suggest that the generation of AAbs binding to this flexible loop of CFH would be a characteristic phenomenon of autoantibody-mediated aHUS.

### Characterization of the binding mode of VHH4 to CCP20

To understand how antibodies, including AAbs, recognize the flexible loop, we characterized the interaction of VHH4 with CCP20 in detail. In the crystal structure, most of antigen–antibody interactions were observed between the CDR3 region of VHH4 and the antigens, not surprising given the importance of the CDR3 loop for antigen recognition of VHHs (35). Sequence analysis using abYsis (36) revealed that among VHHs, the antibody VHH4 possessed a remarkably long CDR3 region consisting of 24 residues (Ala99-Tyr122_VHH4_), remarkably long among VHHs (37).

**Figure 7:**
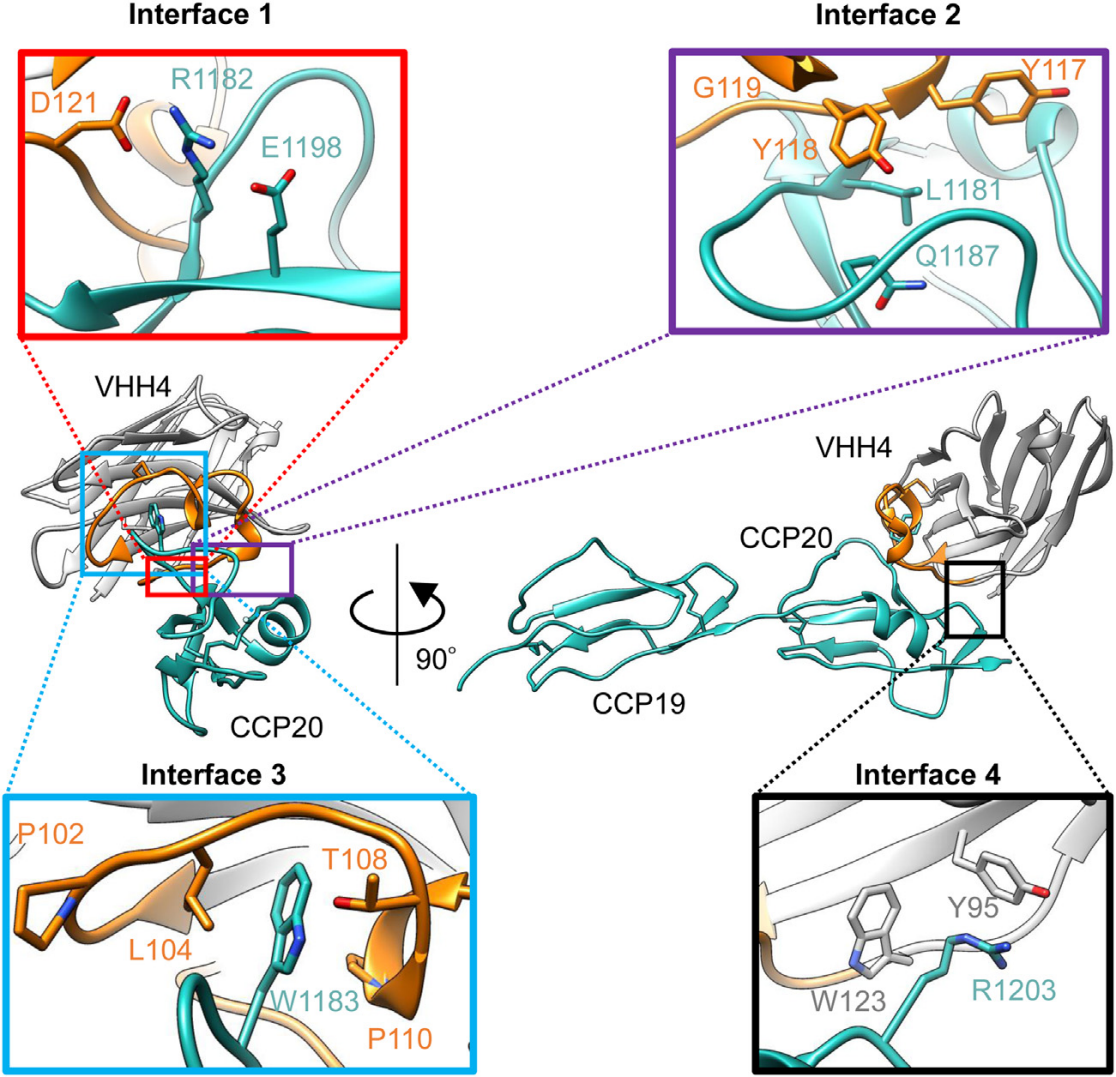
**Structural dissection of the mechanism of recognition.** The figures of the center correspond to the overall structure of the complex of CFH CCP19-20 and VHH4 determined in this work. The *left-center* panel corresponds to a view rotated 90 degrees with respect to the *right-center panel*. The figures in each of the four corners are closeup views of the four interaction surfaces between CCP20 and VHH4 (interface 1–4, respectively). Interface 1 is centered around Arg1182_CCP20_, interface 2 around Leu1181 and Gln1187_CCP20_, interface 3 around Trp1183_CCP20_, and interface 4 around Arg1203_CCP20_. CFH, VHH4 CDR3, and other regions of VHH4 are shown in *light blue*, *orange*, and *gray*, respectively. CCP, complement control protein; CFH, complement factor H.

Two key features regarding the recognition of CFH by VHH4 were found. First, the side chain of Trp1183_CCP20_ was deeply inserted into the long CDR3 of VHH4 (Fig. 7). Consistently, the value of buried surface area (BSA) calculated using PDBePISA was remarkably large (BSA_Trp1183_ = 242.2 Å^2^, Table. S1). Given that the typical recognition mechanisms of VHH antibodies to protein antigens often involve a penetration of the antibody into a cavity of the antigen using their CDR3 loops (38), the antigen recognition mechanism of VHH4 appears to be uncommon for a VHH antibody. Second, an intramolecular disulfide bond in the antibody was formed between Cys106_VHH4_ of the CDR3 and Cys50_VHH4_ of the framework region, thus stabilizing the conformation of the CDR3 loop. The appearance of Cys50_VHH4_ in the framework region is unusual, with a frequency of less than 1% according to the analysis tool abYsis (36). Cys50_VHH4_ might have been selected to stabilize the conformation of the very long CDR3 loop bending toward the framework side, thus fixing the CDR3 loop to a most suitable conformation for the recognition of CFH.

Further, we analyzed the interface between CCP20 and VHH4 in more detail using PDBePISA (Tables. S1 and S2) (39). The interaction area was divided into four regions, interface 1 to interface 4 (Fig. 7). Based on the calculations of the PISA server, in interface 1, a salt bridge was formed between Arg1182_CCP20_ and Asp121_VHH4_. In interface 2, hydrogen bonds between backbone atoms were observed in Leu1181_CCP20_ - Tyr117_VHH4_ and Leu1181_CCP20_ - Gly119_VHH4_ (Fig. 7). Hydrogen bonds between side chains were found between Glu1187_CCP20_ and Tyr118_VHH4_ (Fig. 7). In interface 3, Trp1183_CCP20_ and three residues of VHH4 surrounding Trp1183_CCP20_ (Leu104, Thr108, and Pro110_VHH4_) were predicted to contribute to the interaction. A hydrogen bond was formed between Trp1183_CCP20_ and Thr108_VHH4_ (Fig. 7). Pro102_VHH4_ was also thought to have a role in stabilizing the conformation of the CDR3 of VHH4. In interface 4, Tyr95_VHH4_ and Trp123_VHH4_ were predicted to interact with Arg1203_CCP20_ and a hydrogen bond and a cation–*π* interaction were observed between Tyr95_VHH4_ and Arg1203_CCP20_ (Fig. 7). In summary, numerous interactions stabilize the contact interface between antibody and CFH, explaining the high affinity observed in the binding assay.

### Mutagenesis analyses of interface residues

To verify the relevance of the interactions observed in the crystal structure, we produced the alanine mutant of Trp1183_CCP20_ (W1183A_CCP20_), a residue deeply inserted into the CDR3 loop of VHH4, and subjected it to SPR analyses. The mutant W1183A_CCP20_ showed significantly lower affinity to VHH4 than CFH18-20 WT. The estimated affinity was lower than 10^−6^ M (Fig. S8). W1183A_CCP20_ also showed remarkably lower affinity in the titration experiment by ITC (Fig. S8). These results validated the importance of the recognition of Trp1183_CCP20_ residue by the CDR3 loop as we argued above.

Next, we performed alanine scanning of 10 selected residues mainly belonging to the CDR3 region of VHH4, predicted to be significant for the interaction as inferred from the interaction analyses performed in PDBePISA. We prepared each single alanine mutant and evaluated the contribution of each residue to the molecular recognition of CCP20 by VHH4 using SPR and ITC analyses.

**Figure 8:**
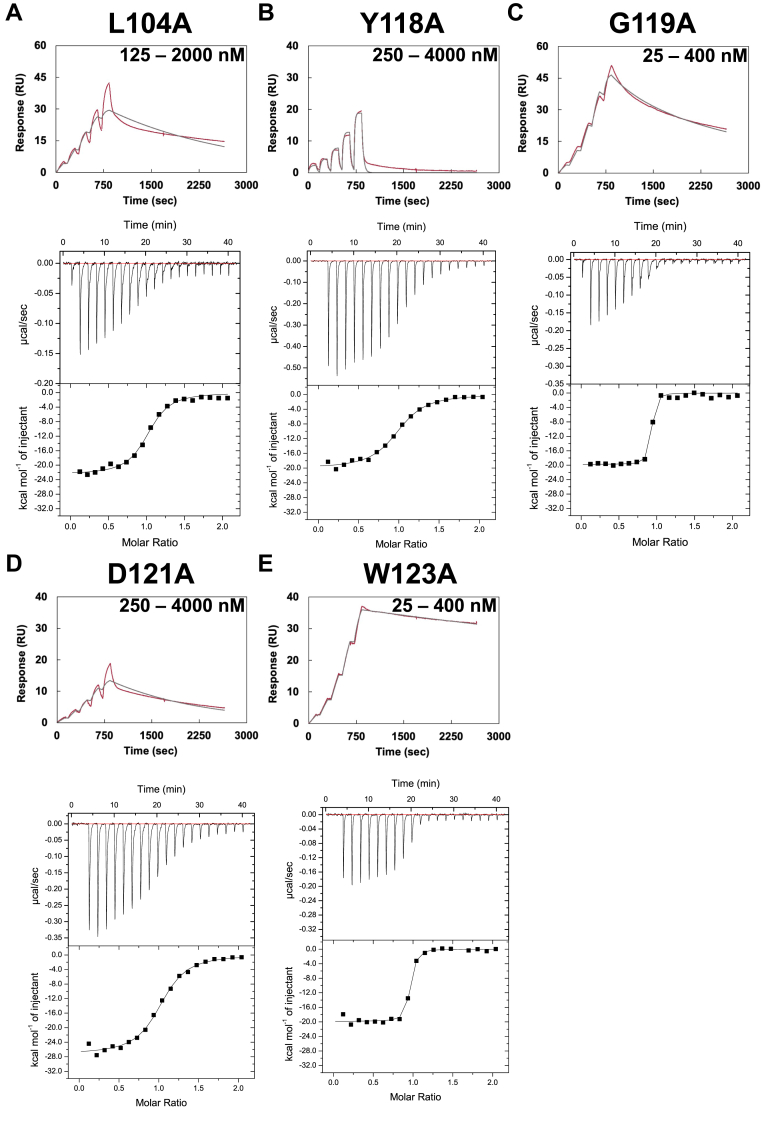
**Mutagenesis analysis of VHH4.***A*–*E*, SPR sensorgram and ITC titration of L104A, Y118A, G119A, D121A, and W123A_CCP20_. *Upper panels* correspond to SPR sensorgrams and *lower panels* to ITC titration curves. SPR measurements were performed in PBS at pH 7.4 supplemented with Tween 20 (0.005%) at 25 °C. CFH was immobilized on a CM5 sensor chip, and VHH4 or its mutants were injected as the analyte. Runs were performed using the single-cycle kinetics method. ITC measurements were conducted in PBS at pH 7.4 at 25 °C. CFH was located into the cell, and VHH4 or alanine-mutants were injected from the syringe. Each titration consisted in 20 injections of VHH4 into the cell containing CFH18-20. Each measurement was performed three times independently. CFH, complement factor H; SPR, surface plasmon resonance.

D121A_VHH4_ in interface 1 showed significant decrease in affinity (*K*_D D121A_/*K*_D WT_ = 7.43 × 10^4^) due to the slower association and much faster dissociation phases than VHH4 WT (Fig. 8*D*, Tables 1 and 2), indicating the importance of Asp121_VHH4_ for VHH4 binding. Although the affinity was significantly reduced by the D121A_VHH4_ mutation, no large loss of enthalpy change compared with WT was observed (ΔΔ*H*_D121A-WT_ = − 0.61 kcal/mol). Considering that Arg1182_CCP20_ engaged in an intramolecular interaction with Glu1198_CCP20_ in the apo form, the heat generated via the direct Arg1182_CCP20_–Asp121_VHH4_ interaction upon the VHH binding to CFH is likely to be composed by loss of Arg1182_CCP20_–Glu1198_CCP20_ interaction. The result showing that no change of binding enthalpy was observed by D121A_VHH4_ mutation also supports that finding.

In interface 2, the affinity significantly decreased (*K*_D Y118A_/*K*_D WT_ = 1.28 × 10^6^) with faster dissociation in the mutant Y118A_VHH4_ (Fig. 8*B*, Tables 1 and 2). In Y118A_VHH4_, a large enthalpy loss compared with VHH4 WT was observed by ITC (ΔΔ*H*_Y118A-WT_ = 9.43 kcal/mol, Fig. 8*B*, Tables 1 and 2), suggesting a large contribution of the hydrogen bond between Tyr118_VHH4_ and Gln1187_CCP20_. Moreover, G119A_VHH4_ showed fast dissociation in SPR (*k*_off G119A_/*k*_off WT_ = 1.10 × 10^4^) and large loss of enthalpy change (ΔΔ*H*_G119A–WT_ = 9.45 kcal/mol) compared with WT (Fig. 8*C* and Table 1). These results indicated the importance of interface 2 for VHH4 binding, and especially Y118A_VHH4_ was identified as the most essential residue in interface 2. In addition, the large enthalpy loss observed in Y118A_VHH4_ and G119A_VHH4_ was unusually large for the loss of a single hydrogen bond.

In interface 3, Pro102, Thr108, and Pro110 _VHH4_ were predicted from the crystal structure to have a large contribution to the interaction (Table S1); however, they showed much smaller changes in kinetic as well as thermodynamic parameters (Figs. S9 and S10, Tables 1 and 2) than Y118A and G119A_VHH4_. On the other hand, L104A_VHH4_ showed low affinity caused by the fast dissociation rate (*K*_D L104A_/*K*_D WT_ = 2.58 × 10^4^, Fig. 8*A*, Tables 1 and 2). In L104A_VHH4_, the small side chain of alanine might generate some space and allow solvent molecules to access the hydrophobic interface, inducing a collapse of the hydrophobic environment. These results suggested that interface 3 displays a single hot spot surrounded by an accumulation of weak interactions like CH–*π* and van der Waals interactions between Trp1183_CCP20_ and residues of the CDR3 including Pro102, Thr108, and Pro110_VHH4_.

In interface 4, W123A_VHH4_ showed fast dissociation by SPR (*k*_off W123A_/*k*_off WT_ = 2.88 × 10^3^, Fig. 8*E* and Table 1) and a significant loss of enthalpy change (ΔΔ*H*_W123A–WT_ = 7.90 kcal/mol) by ITC (Fig. 8*E*, Tables 1 and 2). On the other hand, Y95A_VHH4_ did not show remarkable changes in the binding parameters (Figs. S9 and S10, Tables 1 and 2), despite that this residue forms a hydrogen bond and a cation–*π* interaction with Arg1203_CCP20_ in the crystal structure. Although Trp123_VHH4_ did not form strong direct interactions with CFH like hydrogen bonds or salt bridges, this residue appeared to stabilize the orientation of Arg1203_CCP20_ side chain by van der Waals interactions. The side chain of Arg1203_CCP20_ would strengthen the cation–*π* interactions between Arg1203_CCP20_ and Tyr95_VHH4_.

## Discussion

In this study, we generated a VHH antibody that recognized the C-terminal region of CFH, a major binding site for AAbs in aHUS. Although there is one study reporting the preparation of mAb7968 Fab fragment binding to CCP19 and inhibiting CFH binding to C3b (40), acquisition of anti-CFH VHH antibodies has not been reported. Importantly, the antibody that we termed VHH4 caused hemolysis through inhibition of CFH binding to SA, it did not inhibit the degradation of C3 caused by CFH and CFI, relying on the smaller steric hindrance due to the smaller molecular size of VHH4 than IgG. This VHH will be helpful in further functional analyses of CFH and the elucidation of aHUS pathogenesis.

From the crystal structure and the result of the alanine scan, the interaction surface was divided into four regions from interface 1 to interface 4. In interface 1, taking into consideration that Asp121_VHH4_ is located near the base of the CDR3, interface 1 might play a role not only in forming salt bridges with Arg1182_CCP20_ but also in stabilizing the entire CDR3 of VHH4. In interface 2, decreased affinity and large loss of enthalpy change were observed in Y118A_VHH4_ and G119A_VHH4_. Tyr118_VHH4_ is positioned close to the small helical structure consisting of Gly109–Phe111_VHH4_ in the CDR3 of VHH4. Gly119_VHH4_ is located next to the proximal region of the CDR3 loop surrounding Trp1183_CCP20_. Considering these findings, the role of interface 2 may be stabilizing the CDR3 loop around interface 3 capturing Trp1183_CCP20_, in addition to generating point interactions with CCP20. In interface 3, significant loss of affinity was observed in W1183A_CCP20_, indicating that interactions between Trp1183_CCP20_ and the CDR3 loop surrounding Trp1183_CCP20_ were essential for the molecular recognition by VHH4. Considering that the affinity was significantly decreased in L104A_VHH4_, interface 3 plays a role of surrounding Trp1183_CCP20_ using numerous nonpolar interactions. Finally, interface 4 might play an important role in assisting a tight binding by fixing orientation of the interface between CCP20 and VHH4. As mentioned earlier, VHH4 generated high affinity for the flexible loop consisting of residues Leu1181 to Leu1189_CCP20_ by developing multiple interactions and stabilizing the CDR3 loop conformation through interface 1 to 2, generating interaction surrounding Trp1183_CCP20_ by interface 3, and by forming auxiliary interactions with interface 4.

Our RMSF analyses from the MD simulations indicated that Arg1182–Leu1189_CCP20_ and Arg1210–Arg1215_CCP20_, which are reported as epitopes of anti-CFH AAbs in patients with aHUS, were very flexible regions in CFH18-20. Arnab *et al*. had suggested that Arg1182–Leu1189_CCP20_ was likely to be a flexible region because it appeared in different conformations across several crystal structures including that of the ligand-free CFH18-20, the complex of CFH19-20 and C3d, and the complex of CFH19-20, C3d, and SA (27). In our study, we evaluated the MD of Arg1182–L Leu1189_CCP20_ and Arg1210–Arg1215_CCP20_. In age-related macular degeneration, which is also associated with AAbs against CFH, it has been reported that these AAbs tend to recognize a wide region within the N-terminal domains of CFH (CCP1-8) (41). Considering this precedent, the highly frequency of AAbs recognizing the functional and flexible loops Leu1181–Leu1189 and Arg1210–Arg1215 in CCP20 in the C-terminal domain of CFH may be a characteristic feature of aHUS. Flexibility and dynamical changes of CFH caused by genetic mutations may be involved in the generation of anti-CFH AAbs. Although MD simulations using the complex of anti-CFH Fab and fragment peptide of the epitope have been reported (40, 42), the MD of CFH18-20, in which many pathogenic mutations of aHUS are identified (hot spots), have not been characterized in detail. By comparing the dynamics of CFH with those of CFH-related protein 1 or mutants of CFH, our findings will contribute to elucidate the production mechanisms of anti-CFH AAbs and understanding aHUS pathogenesis.

Herein we have shown that VHH4 did not inhibit the binding of CFH to C3b, but instead it inhibited the binding of CFH to molecules of SA on somatic cells, resulting in hemolysis of sheep erythrocytes. Importantly, VHH4 held Trp1183_CCP20_ side chain into its CDR3 loop resulting in significant conformational changes of this residue with respect to that in the absence of antigen. Given that Trp1183_CCP20_ was suggested to be important for CFH binding to SA based on structural analyses in a previous report (10) and the epitope of VHH4 corresponding to that of anti-CFH AAbs reported before (26, 27), anti-CFH AAbs might adopt similar molecular mechanism to recognize the epitope, leading to pathogenesis of aHUS. Besides, considering that this is the first report describing the structural basis of a recombinant antibody recognizing the same epitope as that of anti-CFH AAbs, our research may serve as a model for CFH recognition by autoantibodies.

In summary, herein we have generated a VHH antibody that recognizes a highly flexible loop in CFH (Arg1182–Leu1189), the same region known to be an epitope of AAbs in aHUS, and characterized the interaction between the antibody and antigen in detail. Our results revealed how the antibody specifically recognizes such a flexible loop. Although further analyses using AAbs from patients will be required to elucidate the molecular mechanism, our results provide important insights to understand how autoantibodies recognize CFH and cause aHUS.

## Experimental procedures

### Expression and purification of recombinant CFH

All CFH constructs were expressed using the same method. The DNA sequence encoding human CFH CCP18-20 with a myc tag and His-tag at the C-terminus for SPR and ITC measurements and with only His-tag at the C-terminus for crystallization were cloned into pcDNA 3.4 vector (A14697, Thermo Fisher Scientific). Recombinant CFH proteins were expressed using Expi293 cells (A14635, Thermo Fisher Scientific) following the manufacturer’s methods. The cells were cultured for 7 days after transfection at 37 °C and 8% CO_2_.

Purification methods were identical for all constructs. The supernatant was collected and filtered followed by dialysis against a solution composed of 20 mM Tris-HCl, pH 8.0, 500 mM NaCl, and 5 mM imidazole. IMAC was conducted using Ni-NTA Agarose (30230, Qiagen). The proteins were eluted with 20 mM Tris-HCl, pH 8.0, 500 mM NaCl, and 200 mM imidazole. The proteins were dialyzed against a solution composed of PBS (pH 7.4). The final purification was performed by SEC using HiLoad 16/60/20 Superdex 75 pg column (28989333, Cytiva) at 4 °C equilibrated in PBS, pH 7.4.

### Circular dichroism

CD spectra of CFH18-20 were collected in a JASCO J-820 instrument using a 1 mM path-length quartz cell. Measurements were carried out at 25 °C in PBS, pH 7.4. Sample concentration was 5 μM. The background (PBS alone) was subtracted, and the spectra were analyzed using spectra manager software (JASCO Co).

### Library construction and selection

Library construction from the peripheral blood B cells obtained from immunized alpaca and antibody selection was conducted as described in a previous study (43). Briefly, total RNA was obtained using Trizol followed by cDNA synthesis. Antibody genes were amplified by PCR and incorporated into a phagemid vector (44). The library DNA was electroporated into *E. coli* XL-1 Blue followed by VCS M13 helper phage infection, and phage production was induced in the presence of 1 mM of isopropyl-1-thio-β-D-galactopyronoside (IPTG). Phage was precipitated from the bacterial supernatant with PEG/NaCl and resuspended in 1% bovine serum albumin/PBS. The VHH antibodies were selected by three rounds of biopanning using microtiter wells, and selected antibody sequences were analyzed.

### Expression and purification of recombinant VHH

All VHH constructs were expressed using the same method. The DNA sequence encoding VHH with His-tag at the C-terminus was cloned into a pRA2 vector (45). VHHs were expressed using BL21 (DE3) *E. coli* (Merck Darmstadt). IPTG (0.5 mM) was added to the growing cells when the *A*_600_ value reached 0.9 ∼1.1 to induce expression of proteins, and overnight culture was harvested. The *E. coli* pellet was resuspended in 20 mM Tris-HCl, pH 8.0, 500 mM NaCl, and 5 mM imidazole, and then the cells were disrupted by sonication. IMAC was conducted using Ni-NTA Agarose (30230, Qiagen). The proteins were eluted with 20 mM Tris-HCl, pH 8.0, 500 mM NaCl, and 200 mM imidazole. The proteins were dialyzed against a solution composed of PBS (pH 7.4). The final purification was performed by SEC using HiLoad 26/60/20 Superdex 75 pg column (28989334, Cytiva) at 4 °C equilibrated in PBS, pH 7.4.

### Expression and purification of recombinant C3d

The DNA sequence encoding C3d was cloned into pET SUMO vector. C3d were expressed using BL21 (DE3) expression system (Merck Darmstadt). To induce expression of proteins, 0.5 mM IPTG was added to the growing cells when the *A*_600_ value reached 0.6. After 16 h at 28 °C, culture was harvested. The *E. coli* pellet was resuspended in 20 mM Tris-HCl, pH 8.0, 500 mM NaCl, and 5 mM imidazole, and then the cells were disrupted by sonication. IMAC was conducted using Ni-NTA Agarose (30230, Qiagen). The proteins were eluted with 20 mM Tris-HCl, pH 8.0, 500 mM NaCl, and 200 mM imidazole. Ulp1 protease was added to cut the His-SUMO tag and dialyzed against a solution composed of 20 mM Tris-HCl, pH 8.0, 500 mM NaCl, and 5 mM imidazole. 2 nd IMAC was conducted to remove the His-SUMO tag and Ulp1 protease and flow through was dialyzed against a solution composed of PBS (pH 7.4) to collect C3d with no Histag. The final purification was performed by SEC using HiLoad 26/60/20 Superdex 75 pg column (28989334, Cytiva) at 4 °C equilibrated in PBS, pH 7.4.

### Mutagenesis of CFH and VHH4

The DNA of each mutant was prepared by site-directed mutagenesis PCR using the KOD-Plus Mutagenesis Kit (SMK101, TOYOBO). The protocol was slightly modified, as we used KOD One PCR Master Mix (KMM-101, TOYOBO) instead of polymerase KOD-Plus for the inverse PCR step. Expression and purification methods of mutants were the same as that of wildtype proteins.

### Surface plasmon resonance

The kinetic parameters of CFH–VHH4 interactions were determined using a Biacore 8K instrument (GE Healthcare). CFH18-20 was immobilized on a CM5 Biacore sensor chip (29149604, GE Healthcare) at around 300 resonance units using the amine-coupling method according to the manufacturer’s methods. After immobilization, VHH4 was injected into the sensor chip at a flow rate of 30 μL/min. The range of each concentration VHH4 was 25 nM, 50 nM, 100 nM, 200 nM, and 400 nM for all the constructs except for the case of W1183A_CCP20_ (625 nM, 1250 nM, 2500 nM, 5000 nM, and 10,000 nM), Y118A_VHH4_, D121A_VHH4_ (250 nM, 500 nM, 1000 nM, 2000 nM, and 4000 nM), and L104A_VHH4_ (125 nM, 250 nM, 500 nM, 1000 nM, and 2000 nM). The association time was 120 s, and the dissociation time was 1800 s. The assays were carried out in PBS, pH 7.4, and Tween 20 (0.005%) at 25 °C. The data were collected by single-cycle kinetics. The data were analyzed with the BIAevaluation software (GE Healthcare).

The interaction analyses between C3d and the complex of CFH18-20 and VHH4 were performed using a Biacore T200 instrument (GE Healthcare). C3d was immobilized on a CM5 Biacore sensor chip at 400 response units using the amine-coupling method according to the manufacturer’s methods. After immobilization, the complex of 5 μM CFH18-20 and VHH4 was injected into the sensor chip at a flow rate of 30 μL/min for 120 s.

### Isothermal titration calorimetry

The thermodynamic parameters of interactions between CFH18-20 and VHH4 were evaluated using an iTC200 microcalorimeter (Malvern Panalytical). Samples were dialyzed against PBS, pH 7.4. CFH18-20 was injected into the cell, and VHH4 was injected into the syringe. The CFH18-20 concentration was 5 μM except for in the case of W1183A_CCP20_ (15 μM), Y118A_VHH4_ (15 μM), and D121A_VHH4_ (10 μM), and the VHH4 concentration was 50 μM except for the case of W1183A_CCP20_ (150 μM), Y118A_VHH4_ (150 μM), and D121A_VHH4_ (100 μM). The thermodynamic parameters were calculated by the fitting of titration curves using ORIGIN 7.0 software (MicroCal).

### Hemolysis assay

Hemolytic assay was performed as previously described (32). SRBCs were purchased from Japan Ram Co. Briefly, 20 μl of pooled citrated plasmas from healthy individuals were incubated with VHH1, VHH4, O72 (mouse anti-human CFH inhibitory antibody binding to CCP18, positive control), or control alpaca antibody against Lysozyme (2b and 2c) at various concentrations, at room temperature for 30 min. The plasma spiked with each antibody was diluted with AP-CFTD buffer (2.5 mM barbital, 1.5 mM sodium barbital, 144 mM NaCl, 7 mM MgCl_2_, and 10 mM EGTA, pH7.2–7.4) to make 100-μl solution, and the mixture was incubated with 100 μl of SRBCs (final concentration, 2.5 × 10^6^ cells/μl) at 37 °C for 30 min. As a blank sample, each plasma was also diluted with AP-CFTD buffer containing 50 mM EDTA and tested in the same way. The reaction with SRBCs was quenched by the addition of 1 ml of VBS-EDTA buffer (2.5 mM barbital, 1.5 mM sodium barbital, 144 mM NaCl, and 2 mM EDTA, pH7.4). After the centrifugation, the absorbance of the supernatant was measured at 414 nm using a plate reader (EnSpire; PerkinElmer). The hemolytic assay was performed in duplicate, and the mean value was shown in the graph. The detailed information of O72 is described in the method of “Cofactor assay in fluid phase”.

### Cofactor assay in fluid phase

Fluid-phase cofactor activity of CFH was measured by using a C3b proteolysis assay (46). To assess the effect of anti-CFH antibody to CFH cofactor activity, C3b (1.0 μg), CFI (0.05 μg), and CFH (0.125 μg) were mixed in the presence of antibody against CFH (1 μg), in a final volume of 20 μl of 10 mM sodium phosphate buffer containing 145 mM NaCl (pH 7.2). The mixture was incubated at 37 °C for 1 h, and the reaction was stopped by the addition of 2 × reducing sample buffer for SDS-PAGE. Alpha-chain of C3b and its cleaving products (68- and 43-kDa fragments of iC3b) were visualized by using Coomassie Brilliant Blue staining. In this assay, two mouse anti-human CFH monoclonal antibodies (O72 and R35) generated in Nara Medical University (32) were used as controls. One is CFH inhibitory antibody (O72) binding to CCP18 at the C-terminus of CFH to inhibit the adhesion of CFH to cell surfaces and induce the enhanced hemolysis of SRBCs in the hemolytic assay, and the other antibody is R35 recognizing both N-terminal and C-terminal fragments of CFH expressed in yeast, which inhibits the fluid-phase cofactor function of CFH, but the hemolysis of SRBCs showed only a slight increase in the hemolytic assay.

### Crystallization of CFH–VHH4 complex

Purified CFH18-20 with His-tag and VHH4 was dialyzed against 20 mM Tris-HCl, pH 8.0, and 200 mM NaCl, respectively. After dialysis, the proteins were mixed to achieve a molar ratio of CFH to VHH4 of 1:1.2 and concentrated to a volume of 5 to 6 ml for SEC purification using Amicon Ultra-15 10K (UFC901024, Merck Millipore). SEC purification was performed using HiLoad 16/60/20 Superdex 75pg column (28989333, Cytiva), in an AKTA system (Cytiva) at 4 °C. After purification, the protein was concentrated to 6 mg/ml (145 μM). Crystallization was conducted using an Oryx8 instrument (Douglas Instruments) using commercial screening kits, PEG/ION 1 (HR2-126, Hampton Research). The crystals were obtained by mixing the complex with a crystallization solution composed of 200 mM sodium phosphate monobasic monohydrate and 20% w/v polyethylene glycol 3350 at 20 °C. Suitable crystals were harvested, incubated in the same solution supplemented with 20% glycerol, and transferred to liquid nitrogen for storage until data collection.

### Data collection and refinement

Diffraction data from a single crystal of CFH-VHH4 complex were collected in beamline AR-NW12A at the Photon Factory under cryogenic conditions (100 K). Diffraction images were processed with the program MOSFLM and merged and scaled with the program SCALA or AIMLESS (47) of CCP4 suite (48). The structure of WT protein was determined by the molecular replacement method using the coordinates of CFH18-20 (PDB entry code 3SW0) (30) and a functionally unrelated VHH (entry code 5LFN) (49) with PHASER (50). The models were refined with the programs REFMACS (51) and built manually with COOT (52). Validation was carried out with PROCHECK (53). Data collection and structure refinement statistics are given in Table 3.

**Table 3:** Data collection and refinement statistics

Data collection	CFH18–20 - VHH
Space group	P 3_2_ 2_2_ 1
Unit cell	
a, b, c (Å)	75.96, 75.96, 143.20
Angles (°)	α = β = 90; γ = 120
Resolution (Å)	48.4–2.60 (2.74–2.60)
Wavelength	1.0000
Reflections (all)	206,520 (30,077)
Reflections (unique)	15,303 (2204)
*R*_*merge*_	0.16 (1.26)
*R*_*p.i.m.*_^a^	0.045 (0.35)
*I/σ (I)*	12.3 (2.6)
Multiplicity	13.5 (13.6)
Completeness (%)	99.9 (99.8)

**Refinement statistics**	

Resolution (Å)	48.4–2.60
*R*_*work*_/*R*_*free*_ (%)	19.1/23.9
No. complex	1
No. atoms	
CFH	1018
VHH	973
Water	25
B-factor (Å^2^)	
CFH	70.5
VHH	66.2
Water	54.6
Ramachandran plot	
Preferred (%)	88.0
Allowed (%)	12.0
Outliers (%)	0
RMSD bond (Å)	0.012
RMSD angle (°)	1.86
Clashscore^a^	3.07 (99th percentile)

Statistical values given in parenthesis refer to the highest resolution bin.

a Reference (61).

### MD simulations

MD simulations of CFH18-20 were performed using GROMACS 2018.3 (54) with the CHARMM36 m force field (55). Protonation state of residdsleues was assessed using PDB2PQR (56). Solvation of structures was performed with TIP3P water (57) in a rectangular box such that the minimum distance to the edge of the box was 15 Å under periodic boundary conditions through the CHARMM-GUI (58). The protein charge was neutralized with added Na or Cl, and additional ions were added to imitate a salt solution of concentration 150 mM.

System was energy-minimized for 5000 steps with the steepest descent algorithm as implemented in GROMACS and equilibrated with the NVT ensemble (298 K) for 1 ns. Further simulations were performed with the NPT ensemble at 298 K for 1000 ns. The time step was set to 2 fs through the MD simulations. A cutoff distance of 12 Å was used for Coulomb and van der Waals interactions. Long-range electrostatic interactions were evaluated using the particle mesh Ewald method (59). Bonds involving hydrogen atoms were constrained by the LINCS algorithm (60). A snapshot was saved every 100 ps. Simulations were conducted three times, each at different initial velocities. All trajectories were analyzed using GROMACS tools. RMSD and RMSF values were computed by rms and rmsf. As the RMSD values were stable after running 50 ns of simulations, we did not consider the first 50 ns when we analyzed the trajectories.

## Data availability

The coordinates and structure factors for the structure of CFH–VHH complex have been deposited in the PDB under entry code 7WKI. All other data are available from the authors upon request. Please send request to Makoto Nakakido, nakakido@g.ecc.u-tokyo.ac.jp.

## Supporting information

This article contains supporting information (32, 62, 63).

## Conflict of interest

The authors declare that they have no conflicts of interest with the contents of this article.

## Supporting information

CFH_Supplementary_forJBC_FinalVer_revise_220416update

## References

[1] Noris M., Remuzzi G. (2009). Atypical hemolytic–uremic syndrome. N. Engl. J. Med..

[2] Raina R., Krishnappa V., Blaha T., Kann T., Hein W., Burke L., Bagga A. (2019). Atypical hemolytic-uremic syndrome: An update on pathophysiology, diagnosis, and treatment. Ther. Apher. Dial..

[3] Meri S. (2016). Self-nonself discrimination by the complement system. FEBS Lett..

[4] Ferreira V.P., Pangburn M.K., Cortés C. (2010). Complement control protein factor H: The good, the bad, and the inadequate. Mol. Immunol..

[5] Rodríguez De Córdoba S., Hidalgo M.S., Pinto S., Tortajada A. (2014). Genetics of atypical hemolytic uremic syndrome (aHUS). Semin. Thromb. Hemost..

[6] Rodriguez E., Rallapalli P.M., Osborne A.J., Perkins S.J. (2014). New functional and structural insights from updated mutational databases for complement factor H, Factor I, membrane cofactor protein and C3. Biosci. Rep..

[7] Wu J., Wu Y.Q., Ricklin D., Janssen B.J.C., Lambris J.D., Gros P. (2009). Structure of complement fragment C3b-factor H and implications for host protection by complement regulators. Nat. Immunol..

[8] Schmidt C.Q., Herbert A.P., Hocking H.G., Uhrín D., Barlow P.N. (2008). Translational mini-review series on complement factor H: Structural and functional correlations for factor H. Clin. Exp. Immunol..

[9] Morgan H.P., Schmidt C.Q., Guariento M., Blaum B.S., Gillespie D., Herbert A.P., Kavanagh D., Mertens H.D.T., Svergun D.I., Johansson C.M., Uhrín D., Barlow P.N., Hannan J.P. (2011). Structural basis for engagement by complement factor H of C3b on a self surface. Nat. Struct. Mol. Biol..

[10] Blaum B.S., Hannan J.P., Herbert A.P., Kavanagh D., Uhrín D., Stehle T. (2015). Structural basis for sialic acid-mediated self-recognition by complement factor H. Nat. Chem. Biol..

[11] Kerr H., Wong E., Makou E., Yang Y., Marchbank K., Kavanagh D., Richards A., Herbert A.P., Barlow P.N. (2017). Disease-linked mutations in factor H reveal pivotal role of cofactor activity in Self-surface–selective regulation of complement activation. J. Biol. Chem..

[12] Schmidt C.Q., Herbert A.P., Kavanagh D., Gandy C., Fenton C.J., Blaum B.S., Lyon M., Uhrín D., Barlow P.N. (2008). A new map of glycosaminoglycan and C3b binding sites on factor H. J. Immunol..

[13] Meri S., Pangburn M.K. (1990). Discrimination between activators and nonactivators of the alternative pathway of complement: Regulation via a sialic acid/polyanion binding site on factor H. Proc. Natl. Acad. Sci. U. S. A..

[14] Dragon-Durey M.A., Loirat C., Cloarec S., Macher M.A., Blouin J., Nivet H., Weiss L., Fridman W.H., Frémeaux-Bacchi V. (2005). Anti-factor H autoantibodies associated with atypical hemolytic uremic syndrome. J. Am. Soc. Nephrol..

[15] Józsi M., Licht C., Strobel S., Zipfel S.L.H., Richter H., Heinen S., Zipfel P.F., Skerka C. (2008). Factor H autoantibodies in atypical hemolytic uremic syndrome correlate with CFHR1/CFHR3 deficiency. Blood.

[16] Hofer J., Giner T., Józsi M. (2014). Complement factor h-antibody-associated hemolytic uremic syndrome: Pathogenesis, clinical presentation, and treatment. Semin. Thromb. Hemost..

[17] Józsi M., Strobel S., Dahse H.M., Liu W.S., Hoyer P.F., Oppermann M., Skerka C., Zipfel P.F. (2007). Anti-factor H autoantibodies block C-terminal recognition function of factor H in hemolytic uremic syndrome. Blood.

[18] Moore I., Strain L., Pappworth I., Kavanagh D., Barlow P.N., Herbert A.P., Schmidt C.Q., Staniforth S.J., Holmes L.V., Ward R., Morgan L., Goodship T.H.J., Marchbank K.J. (2010). Association of factor H autoantibodies with deletions of CFHR1, CFHR3, CFHR4, and with mutations in CFH, CFI, CD46, and C3 in patients with atypical hemolytic uremic syndrome. Blood.

[19] Strobel S., Hoyer P.F., MacHe C.J., Sulyok E., Liu W.S., Richter H., Oppermann M., Zipfel P.F., Józsi M. (2010). Functional analyses indicate a pathogenic role of factor H autoantibodies in atypical haemolytic uraemic syndrome. Nephrol. Dial. Transpl..

[20] Blanc C., Roumenina L.T., Ashraf Y., Hyvärinen S., Sethi S.K., Ranchin B., Niaudet P., Loirat C., Gulati A., Bagga A., Fridman W.H., Sautès-Fridman C., Jokiranta T.S., Frémeaux-Bacchi V., Dragon-Durey M.-A. (2012). Overall neutralization of complement factor H by autoantibodies in the acute phase of the autoimmune form of atypical hemolytic uremic syndrome. J. Immunol..

[21] Xue X., Wu J., Ricklin D., Forneris F., Di Crescenzio P., Schmidt C.Q., Granneman J., Sharp T.H., Lambris J.D., Gros P. (2017). Regulator-dependent mechanisms of C3b processing by factor i allow differentiation of immune responses. Nat. Struct. Mol. Biol..

[22] Foltyn Zadura A., Zipfel P.F., Bokarewa M.I., Sturfelt G., Jönsen A., Nilsson S.C., Hillarp A., Saxne T., Trouw L.A., Blom A.M. (2012). Factor H autoantibodies and deletion of Complement Factor H-Related protein-1 in rheumatic diseases in comparison to atypical hemolytic uremic syndrome. Arthritis Res. Ther..

[23] Abarrategui-Garrido C., Martínez-Barricarte R., López-Trascasa M., Rodríguez De Córdoba S., Sánchez-Corral P. (2009). Characterization of complement factor H-related (CFHR) proteins in plasma reveals novel genetic variations of CFHR1 associated with atypical hemolytic uremic syndrome. Blood.

[24] Dragon-Durey M.A., Blanc C., Marliot F., Loirat C., Blouin J., Sautes-Fridman C., Fridman W.H., Frémeaux-Bacchi V. (2009). The high frequency of complement factor H related CFHR1 gene deletion is restricted to specific subgroups of patients with atypical haemolytic uraemic syndrome. J. Med. Genet..

[25] Strobel S., Abarrategui-Garrido C., Fariza-Requejo E., Seeberger H., Sánchez-Corral P., Józsi M. (2011). Factor H-related protein 1 neutralizes anti-factor H autoantibodies in autoimmune hemolytic uremic syndrome. Kidney Int..

[26] Trojnár E., Józsi M., Uray K., Csuka D., Szilágyi Á., Milosevic D., Stojanovic V.D., Spasojevic B., Rusai K., Müller T., Arbeiter K., Kelen K., Szabó A.J., Reusz G.S., Hyvärinen S. (2017). Analysis of linear antibody epitopes on factor H and CFHR1 using sera of patients with autoimmune atypical hemolytic uremic syndrome. Front. Immunol..

[27] Bhattacharjee A., Reuter S., Trojnár E., Kolodziejczyk R., Hyvärinen H.S.S., Uzonyi B., Szilágyi Á., Prohászka Z., Goldman A., Józsi M., Jokiranta T.S. (2015). The major autoantibody epitope on factor H in atypical hemolytic uremic syndrome is structurally different from its homologous site in factor H-related protein 1, supporting a novel model for induction of autoimmunity in this disease. J. Biol. Chem..

[28] Makou E., Herbert A.P., Barlow P.N. (2015). Creating functional sophistication from simple protein building blocks, exemplified by factor H and the regulators of complement activation. Biochem. Soc. Trans..

[29] Meri T., Amdahl H., Lehtinen M.J., Hyvärinen S., McDowell J.V., Bhattacharjee A., Meri S., Marconi R., Goldman A., Jokiranta T.S. (2013). Microbes bind complement inhibitor factor H via a common site. PLoS Pathog..

[30] Morgan H.P., Mertens H.D.T., Guariento M., Schmidt C.Q., Soares D.C., Svergun D.I., Herbert A.P., Barlow P.N., Hannan J.P. (2012). Structural analysis of the C-terminal region (modules 18-20) of complement regulator factor H (FH). PLoS One.

[31] Prosser B.E., Johnson S., Roversi P., Herbert A.P., Blaum B.S., Tyrrell J., Jowitt T.A., Clark S.J., Tarelli E., Uhrín D., Barlow P.N., Sim R.B., Day A.J., Lea S.M. (2007). Structural basis for complement factor H-linked age-related macular degeneration. J. Exp. Med..

[32] Yoshida Y., Miyata T., Matsumoto M., Shirotani-Ikejima H., Uchida Y., Ohyama Y., Kokubo T., Fujimura Y. (2015). A novel quantitative hemolytic assay coupled with restriction fragment length polymorphisms analysis enabled early diagnosis of atypical hemolytic uremic syndrome and identified unique predisposing mutations in Japan. PLoS One.

[33] Uversky V.N., Van Regenmortel M.H.V. (2021). Mobility and disorder in antibody and antigen binding sites do not prevent immunochemical recognition. Crit. Rev. Biochem. Mol. Biol..

[34] Goh G.K.M., Dunker A.K., Foster J.A., Uversky V.N. (2019). Hiv vaccine mystery and viral shell disorder. Biomolecules.

[35] Zavrtanik U., Lukan J., Loris R., Lah J., Hadži S. (2018). Structural basis of epitope recognition by heavy-chain camelid antibodies. J. Mol. Biol..

[36] Swindells M.B., Porter C.T., Couch M., Hurst J., Abhinandan K.R., Nielsen J.H., Macindoe G., Hetherington J., Martin A.C.R. (2017). abYsis: Integrated antibody sequence and structure—management, analysis, and prediction. J. Mol. Biol..

[37] Al Qaraghuli M.M., Ferro V.A. (2017). Analysis of the binding loops configuration and surface adaptation of different crystallized single-domain antibodies in response to various antigens. J. Mol. Recognit..

[38] Henry K.A., MacKenzie C.R. (2018). Antigen recognition by single-domain antibodies: Structural latitudes and constraints. MAbs.

[39] Krissinel E., Henrick K. (2007). Inference of macromolecular assemblies from crystalline state. J. Mol. Biol..

[40] Bushey R.T., Moody M.A., Nicely N.L., Haynes B.F., Alam S.M., Keir S.T., Bentley R.C., Roy Choudhury K., Gottlin E.B., Campa M.J., Liao H.X., Patz E.F. (2016). A therapeutic antibody for cancer, derived from single human B cells. Cell Rep..

[41] Dhillon B., Wright A.F., Tufail A., Pappworth I., Hayward C., Moore I., Strain L., Kavanagh D., Barlow P.N., Herbert A.P., Schmidt C.Q., Armbrecht A.M., Laude A., Deary I.J., Staniforth S.J. (2010). Complement factor H autoantibodies and age-related macular degeneration. Invest. Ophthalmol. Vis. Sci..

[42] Yang B., Lin S.J., Ren J.Y., Liu T., Wang Y.M., Li C.M., Xu W.W., He Y.W., Zheng W.H., Zhao J., Yuan X.H., Liao H.X. (2019). Molecular docking and molecular dynamics (MD) simulation of human anti-complement factor h (CFH) antibody Ab42 and CFH polypeptide. Int. J. Mol. Sci..

[43] Ishii M., Nakakido M., Caaveiro J.M.M., Kuroda D., Okumura C.J., Maruyama T., Entzminger K., Tsumoto K. (2021). Structural basis for antigen recognition by methylated lysine-specific antibodies. J. Biol. Chem..

[44] Barbas C.F., Burton D.R., Scott J.K., Silverman G.J. (2001).

[45] Makabe K., Asano R., Ito T., Tsumoto K., Kudo T., Kumagai I. (2005). Tumor-directed lymphocyte-activating cytokines: Refolding-based preparation of recombinant human interleukin-12 and an antibody variable domain-fused protein by additive-introduced stepwise dialysis. Biochem. Biophys. Res. Commun..

[46] Pechtl I.C., Kavanagh D., Mcintosh N., Harris C.L., Barlow P.N. (2011). Disease-associated N-terminal complement factor H mutations perturb cofactor and decay-accelerating activities. J. Biol. Chem..

[47] Evans P. (2006). Scaling and assessment of data quality. Acta Crystallogr. Sect. D Biol. Crystallogr..

[48] Winn M.D., Ballard C.C., Cowtan K.D., Dodson E.J., Emsley P., Evans P.R., Keegan R.M., Krissinel E.B., Leslie A.G.W., McCoy A., McNicholas S.J., Murshudov G.N., Pannu N.S., Potterton E.A., Powell H.R. (2011). Overview of the CCP4 suite and current developments. Acta Crystallogr. Sect. D Biol. Crystallogr..

[49] Kromann-Hansen T., Louise Lange E., Peter Sørensen H., Hassanzadeh-Ghassabeh G., Huang M., Jensen J.K., Muyldermans S., Declerck P.J., Komives E.A., Andreasen P.A. (2017). Discovery of a novel conformational equilibrium in urokinase-type plasminogen activator. Sci. Rep..

[50] McCoy A.J., Grosse-Kunstleve R.W., Adams P.D., Winn M.D., Storoni L.C., Read R.J. (2007). Phaser crystallographic software. J. Appl. Crystallogr..

[51] Murshudov G.N., Vagin A.A., Dodson E.J. (1997). Refinement of macromolecular structures by the maximum-likelihood method. Acta Crystallogr. Sect. D Biol. Crystallogr..

[52] Emsley P., Lohkamp B., Scott W.G., Cowtan K. (2010). Features and development of coot. Acta Crystallogr. Sect. D Biol. Crystallogr..

[53] Laskowski R.A., MacArthur M.W., Moss D.S., Thornton J.M. (1993). Procheck: A program to check the stereochemical quality of protein structures. J. Appl. Crystallogr..

[54] Abraham M.J., Murtola T., Schulz R., Páll S., Smith J.C., Hess B., Lindah E. (2015). Gromacs: High performance molecular simulations through multi-level parallelism from laptops to supercomputers. SoftwareX.

[55] Huang J., Rauscher S., Nawrocki G., Ran T., Feig M., De Groot B.L., Grubmüller H., MacKerell A.D. (2016). CHARMM36m: An improved force field for folded and intrinsically disordered proteins. Nat. Methods.

[56] Dolinsky T.J., Nielsen J.E., McCammon J.A., Baker N.A. (2004). PDB2PQR: An automated pipeline for the setup of Poisson-Boltzmann electrostatics calculations. Nucl. Acids Res..

[57] Jorgensen W.L., Chandrasekhar J., Madura J.D., Impey R.W., Klein M.L. (1983). Comparison of simple potential functions for simulating liquid water. J. Chem. Phys..

[58] Jo S., Kim T., Iyer V., Im W. (2008). CHARMM-GUI: A web-based graphical user interface for CHARMM. J. Comput. Chem..

[59] Darden T., York D., Pedersen L. (1993). Particle mesh Ewald: An N·log(N) method for Ewald sums in large systems. J. Chem. Phys..

[60] Hess B., Bekker H., Berendsen H.J.C., Fraaije J.G.E.M. (1997). LINCS: A linear constraint solver for molecular simulations. J. Comput. Chem..

[61] Williams C.J., Headd J.J., Moriarty N.W., Prisant M.G., Videau L.L., Deis L.N., Verma V., Keedy D.A., Hintze B.J., Chen V.B., Jain S., Lewis S.M., Arendall W.B., Snoeyink J., Adams P.D. (2018). MolProbity: More and better reference data for improved all-atom structure validation. Protein Sci..

[62] Sharon K.M., Thomas J.J., Nicholas P.C. (2005). How to study proteins by circular dichroism. Biochim. Biophys. Acta.

[63] Akiba H., Tamura H., Kiyoshi M., Yanaka S., Sugase K., Caaveiro J.M.M., Tsumoto K. (2019). Structural and thermodynamic basis for the recognition of the substrate-binding cleft on hen egg lysozyme by a single-domain antibody. Sci. Rep..

