# Supplementary material for: Antibody recognition of complement factor H reveals a flexible loop involved in atypical hemolytic uremic syndrome pathogenesis
Source: J Biol Chem. 2022 Apr 20;298(6):101962. doi: 10.1016/j.jbc.2022.101962 (PMC9127587; doi:10.1016/j.jbc.2022.101962)
Supplement: CFH_Supplementary_forJBC_FinalVer_revise_220416update [file mmc1.docx]

Supplementary Information


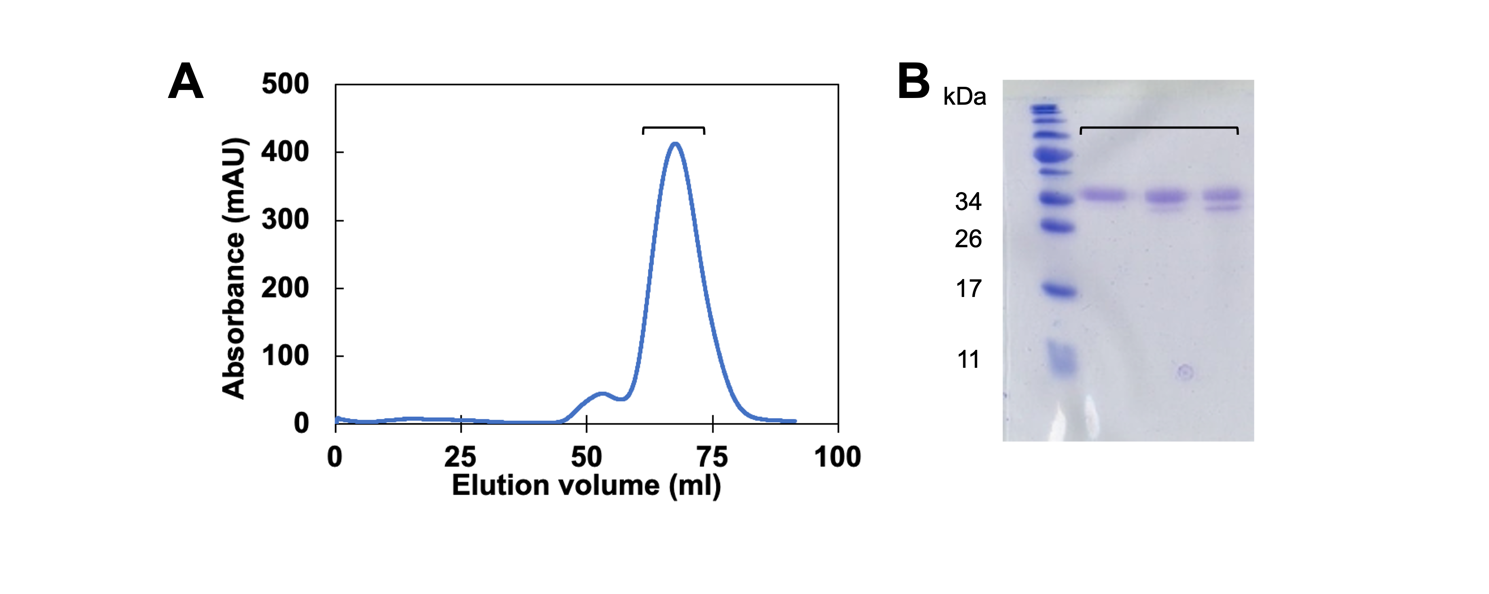


**Figure. S1 Purification of CFH18-20.** (A) SEC profile of CFH18-20. (B) SDS-PAGE and CBB staining of CFH18-20 after SEC. The upper band corresponded to CFH18-20 with glycosylation and the lower band corresponded to CFH18-20 without glycosylation.


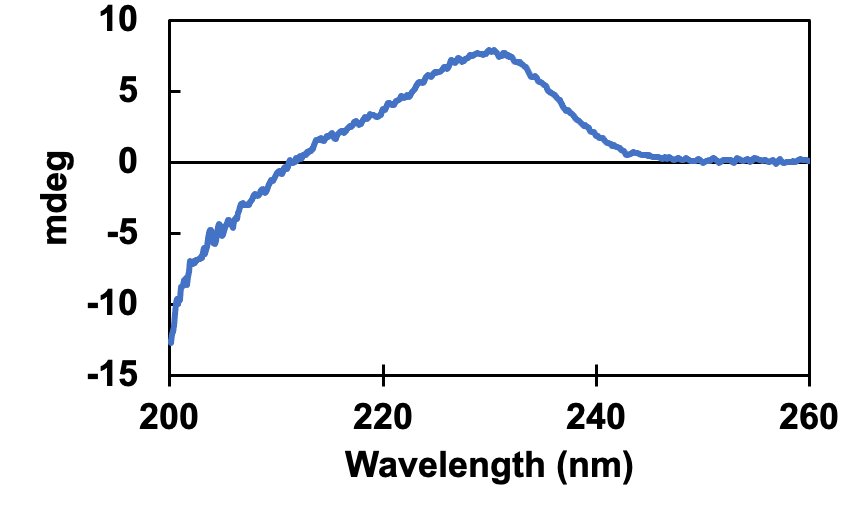


**Figure S2. CD spectrum of CFH18-20.** Measurement was performed at 25 $^{\circ}C$ in PBS pH 7.4. Peaks corresponding to regular $\alpha$-helix (208~209 nm and 222 nm) or $\beta$-sheet (216~218 nm) were not observed.


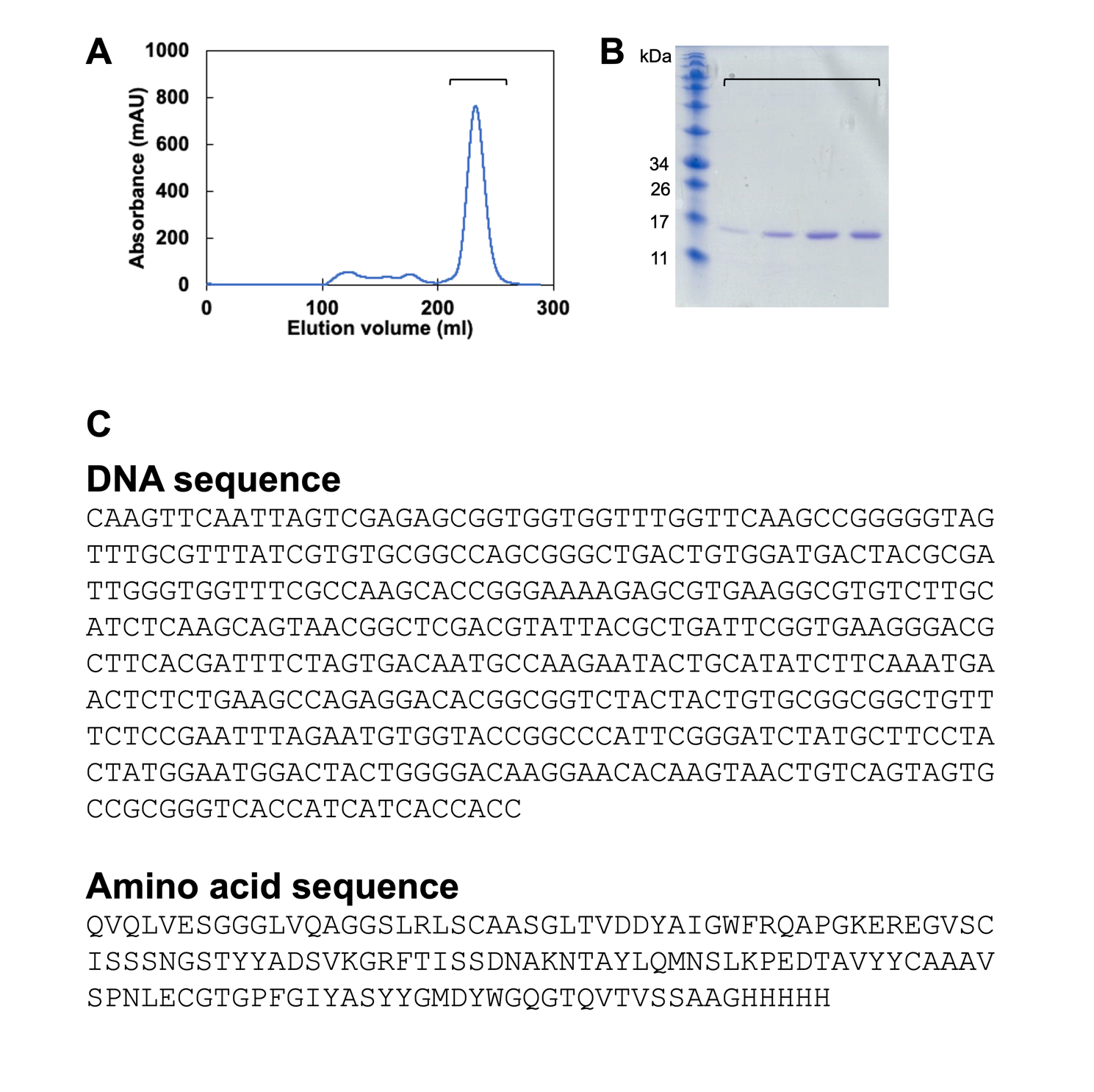


**Figure S3. Purification and sequence information of VHH4.** (A) SEC profile of VHH4. (B) SDS-PAGE and CBB staining of VHH4 after SEC. (C) DNA and amino acid sequence of VHH4.


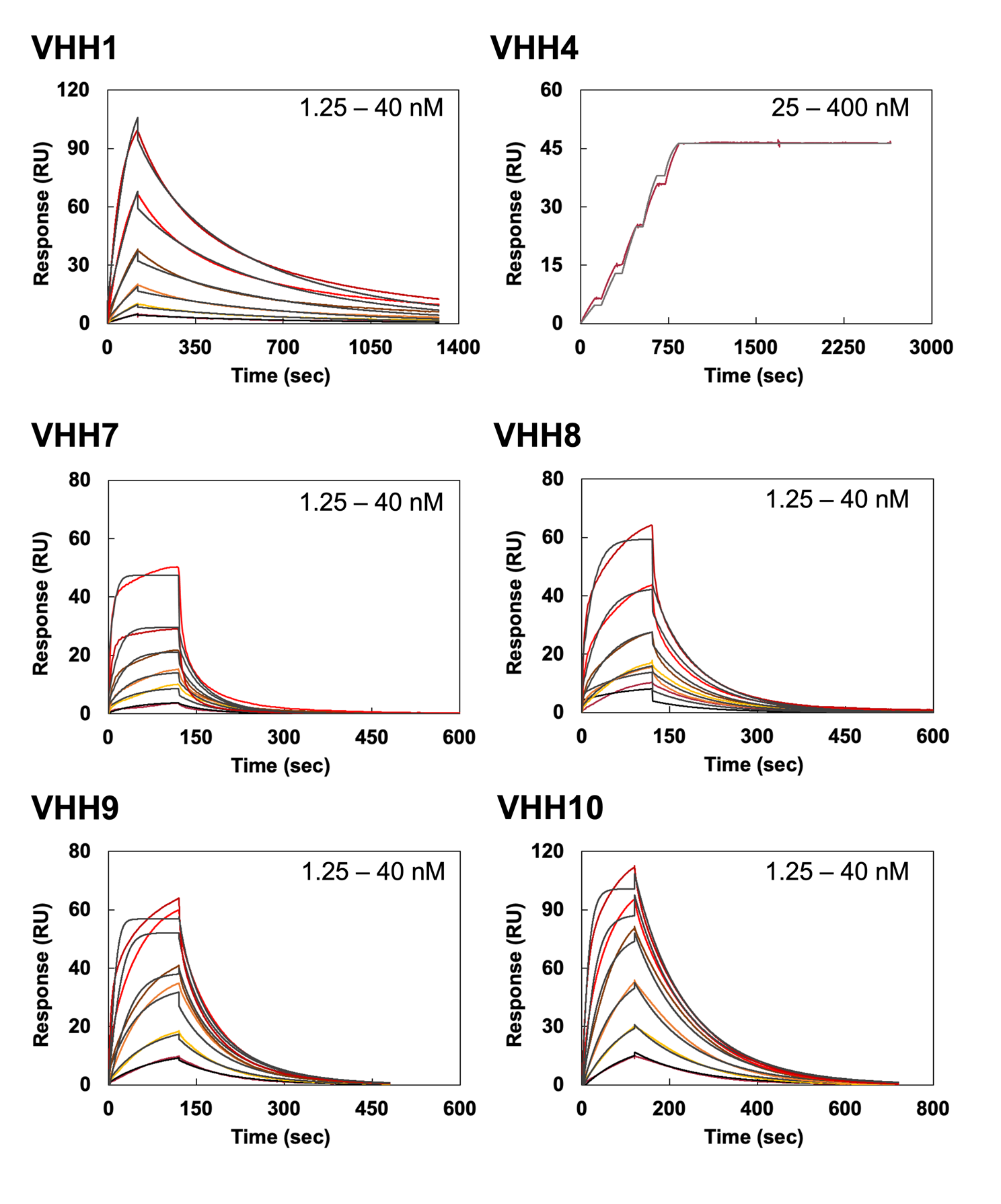


**Figure S4. Binding of various VHHs to CFH18-20 by SPR.** Measurements were performed at 25 $^{\circ}$C in PBS pH 7.4 supplemented with 0.005 % Tween20. CFH18-20 was immobilized on a CM5 sensor chip and VHH4 was injected as the analyte, at the concentration range indicated in each panel. Measurements of the other VHHs were conducted using multi kinetics method, except that of VHH4, which was performed using single kinetics method because of the difficulty to regenerate the surface due to the high affinity of the antibody for CFH18-20.


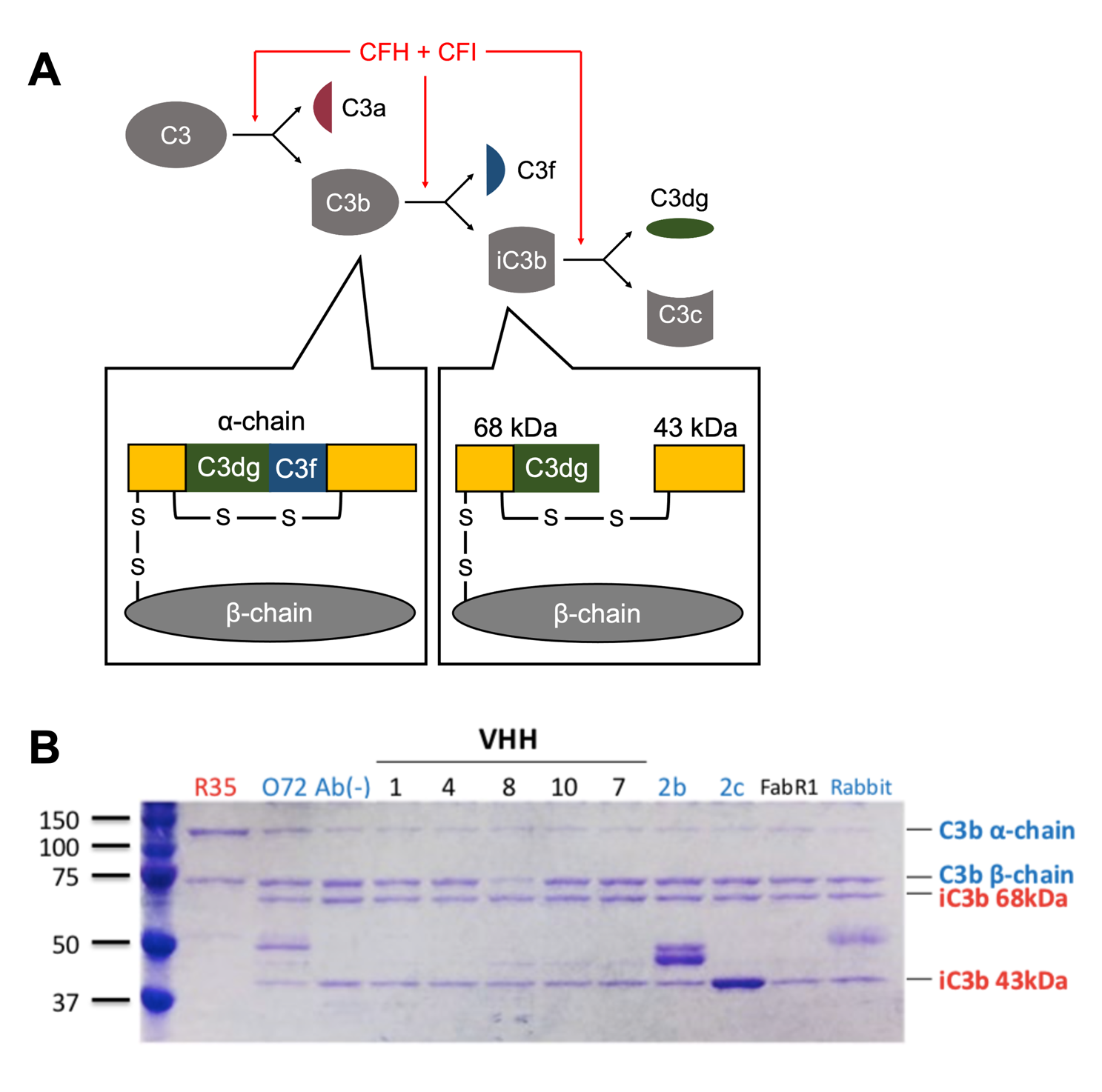


**Figure S5. Cofactor analyses of VHH4.** (A) Schematic diagram of the decomposition cascade reaction of C3. (B) CBB staining of samples. R35 is an IgG antibody used as a positive control for fluid-phase cofactor assay. O72 is a different IgG that recognizes CCP18 of CFH resulting in hemolysis of sheep red blood cells (2). VHHs, 1, 7, 8, and 10 are anti-CFH VHH antibodies that recognize regions other than that recognized by VHH4. 2b and 2c are alpaca anti lysozyme D3L11 heavy chain antibodies used as negative control (3). FabR1 is an anti-CFH antibody that recognizes CCP18. Rabbit antibody is IgG antibody used as negative control.


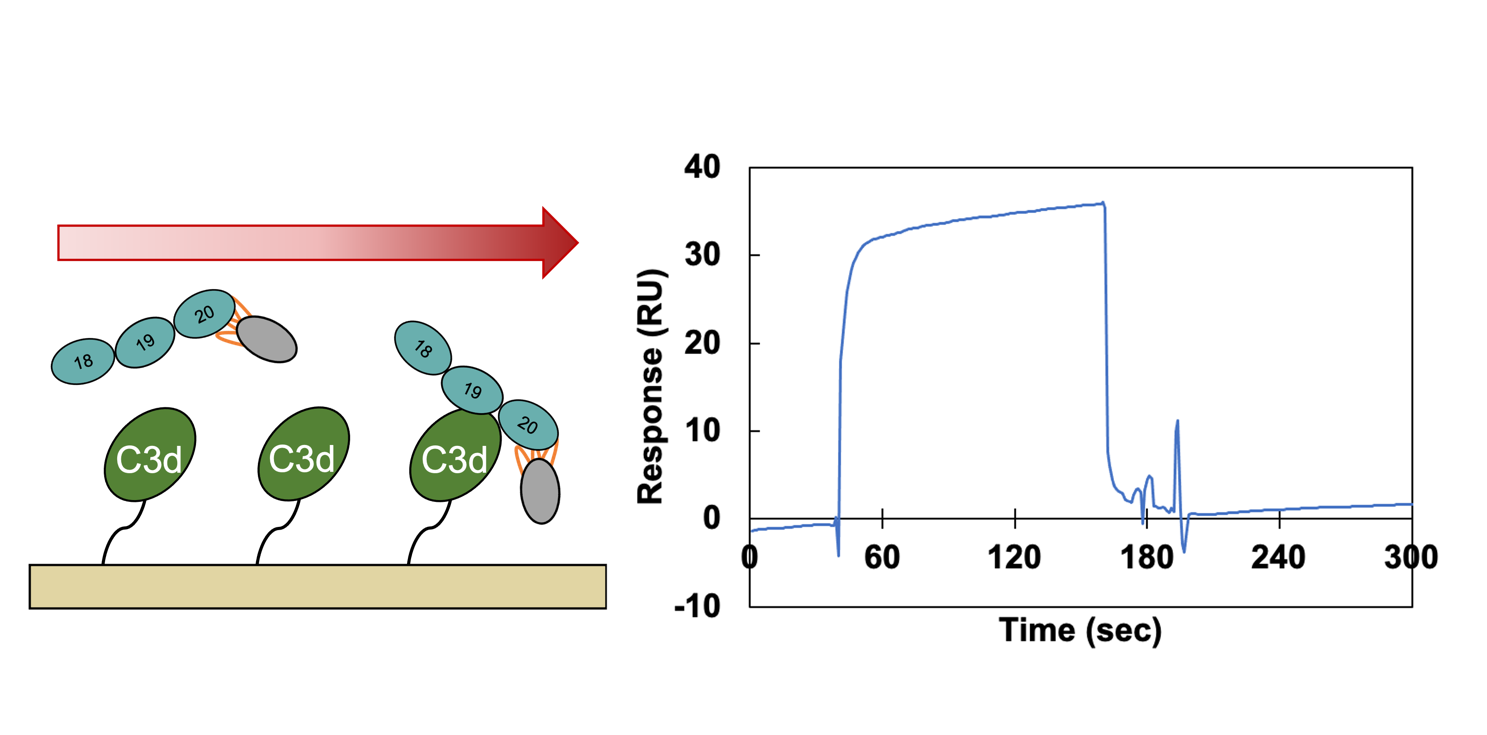


Figure. S6. Interaction analyses between C3d and the complex of CFH18-20 and VHH4 by SPR. Measurement was performed at 25 $^{\circ}$C in PBS pH 7.4 and 0.005 % Tween20. Recombinant C3d were immobilized on the CM5 sensor chip and the complex of CFH18-20 and VHH4 were injected as the analyte at the concentration of 5 $\mu$M.


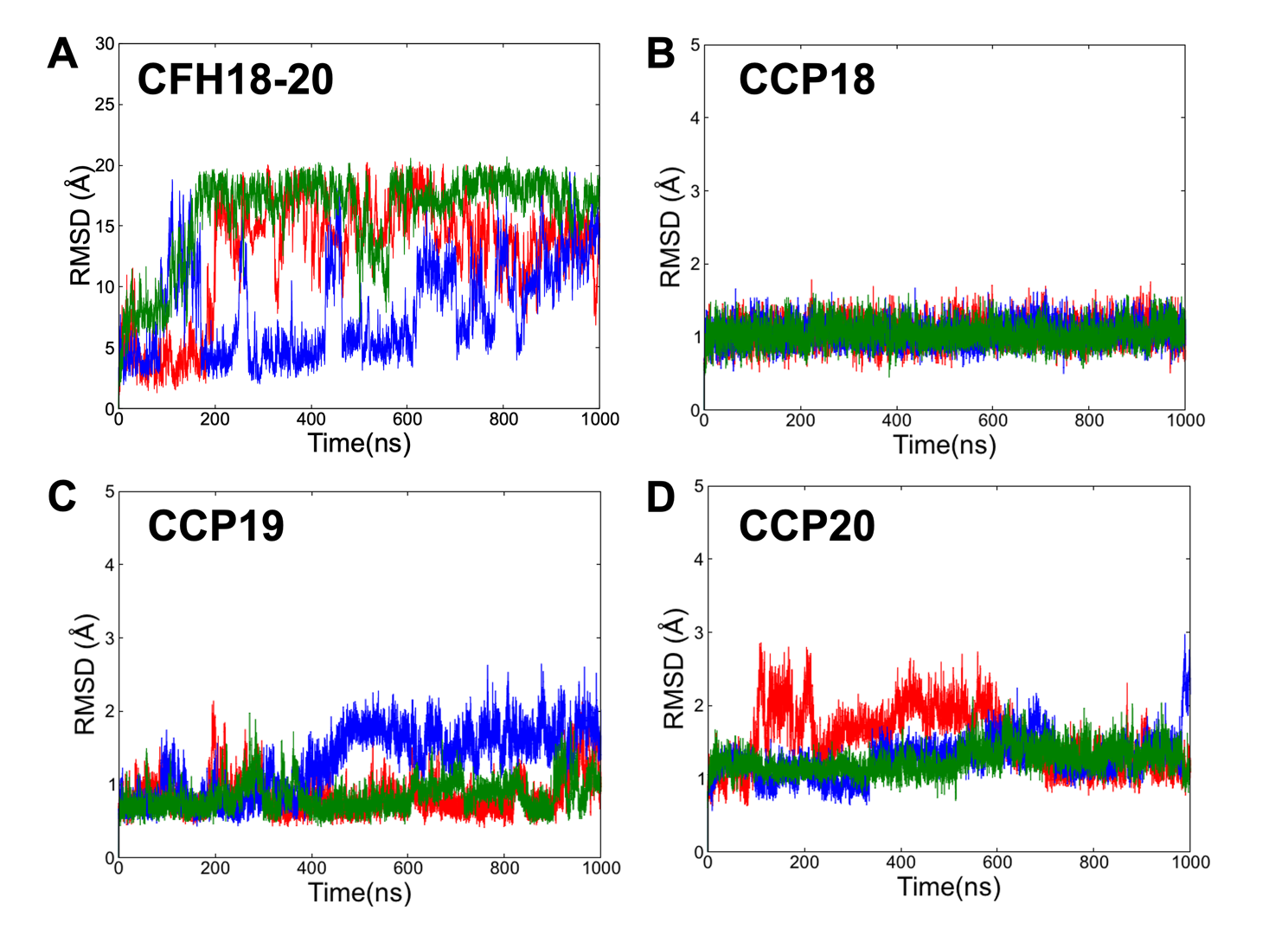


**Figure S7. MD simulations of CFH18-20. RMSD of C**$\boldsymbol{\alpha}$ **atoms in CFH18-20 and each domain were separately calculated.** (A) RMSD calculated using C$\alpha$ atoms in CFH18-20. (B) C$\alpha$ atoms in CCP18. (C) C$\alpha$ atoms in CCP19. (D) C$\alpha$ atoms in CCP20. Three independent runs were performed. The graphs colored in red, blue, and green were calculated based on the first, second, and third run, respectively.


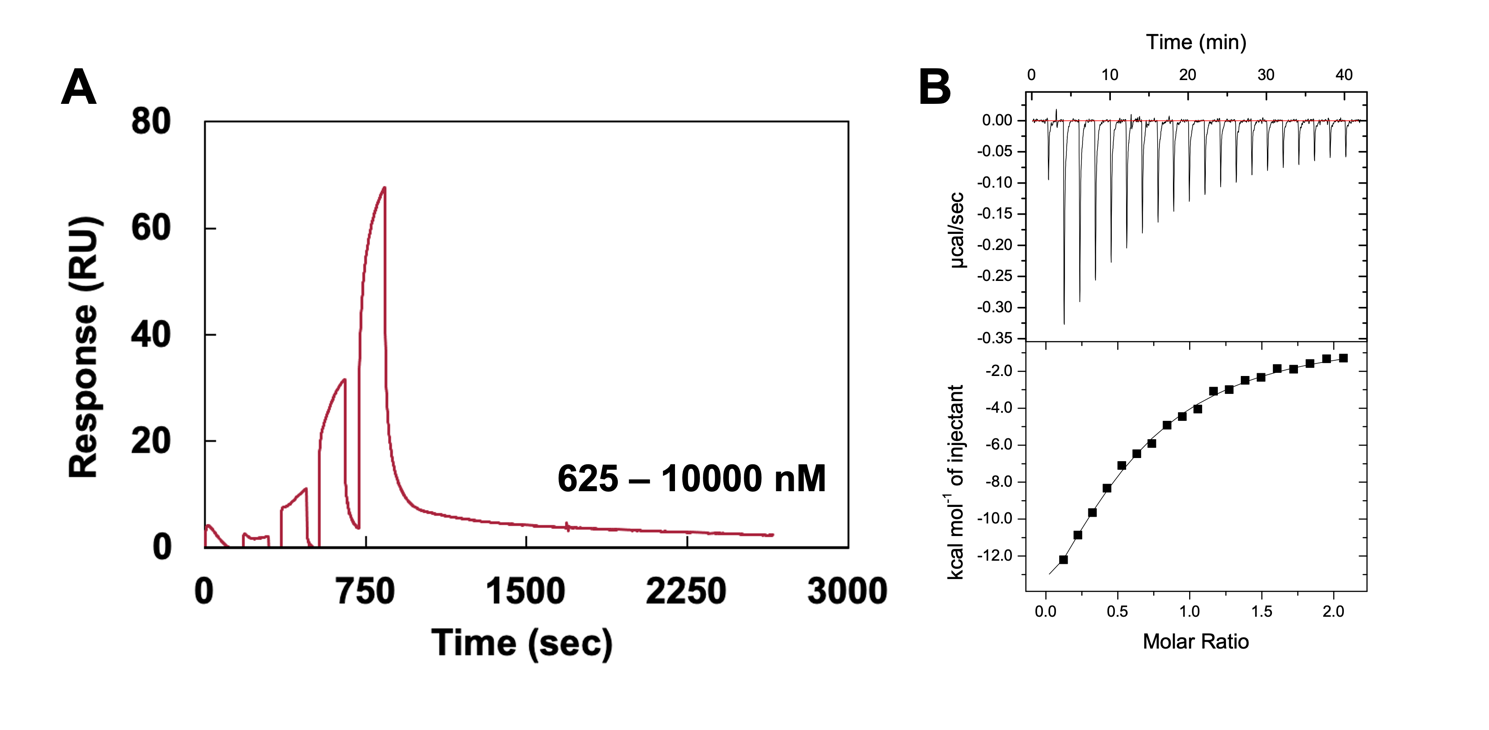


**Figure S8. Binding of VHH4 to W11183A_CCP20_. (A) SPR sensorgram and (B) ITC data of interaction between W1183A_CCP20_ and VHH4 WT.** SPR measurement was performed at 25 $^{\circ}$C in PBS pH 7.4 and 0.005 % Tween20. W1183A_CCP20_ was immobilized on a CM5 sensor chip and VHH4 was injected as the analyte. In ITC, the measurement was conducted in PBS pH 7.4. Remarkable affinity loss was observed in this mutation. Although binding was detected, the dissociation constant and other parameters could not be determined.


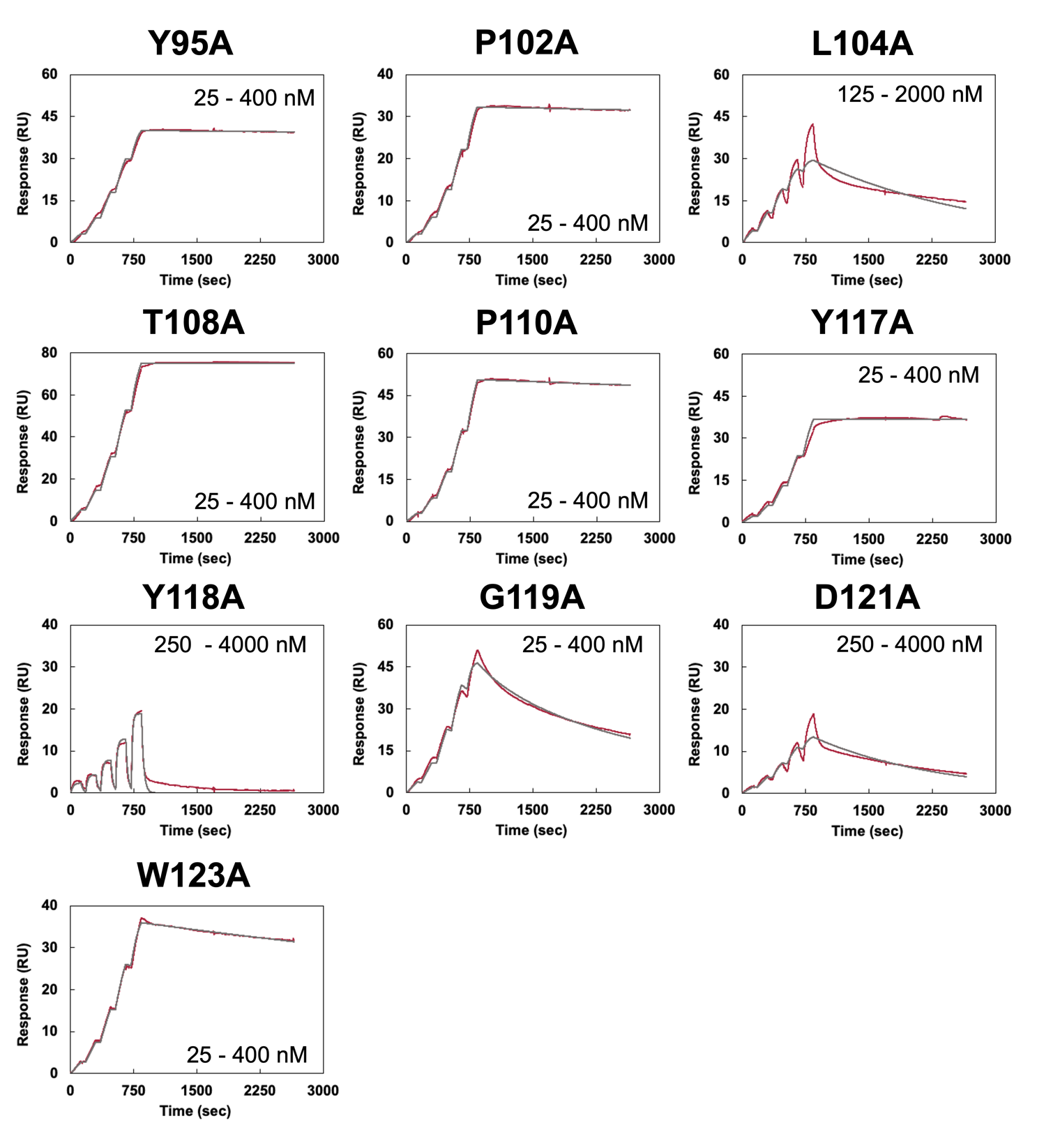


**Figure S9. Alanine scanning and binding of each mutant of VHHs to immobilized CFH18-20 by SPR.** Measurements were conducted by single kinetics method in PBS pH 7.4 and 0.005 % Tween 20 at 25 $^{\circ}$C. CFH18-20 was immobilized on a CM5 sensor chip using the amine coupling method. Mutations in VHH4 were indicated in each panel. Experimental data and fitting are represented with the red and black lines, respectively. The kinetic parameters from the fittings of the SPR data are shown in Table 1.


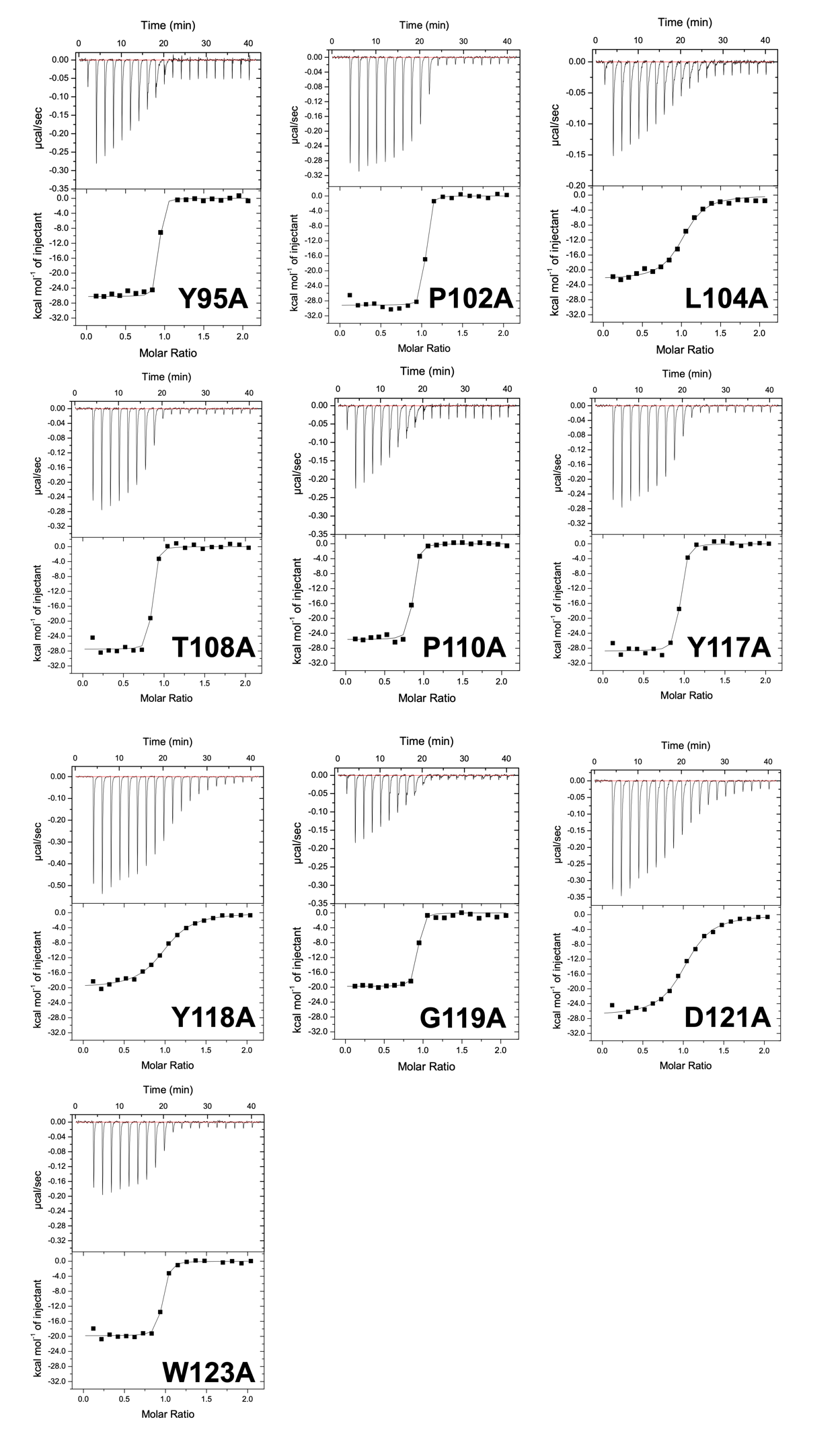


**Figure S10. Alanine scan of VHH4 monitored by ITC.** Measurements were performed in PBS pH 7.4 at 25 $^{\circ}$C. CFH18-20 was placed in cell and the VHH4 alanine mutants in the syringe. 20 separate injections were employed in each measurement. The concentration of CFH18-20 was 5 $\mu$M, except for the titration of Y118A (15 $\mu$M) and D121A (10 $\mu$M). The concentration of VHH4 was 50 $\mu M$, except for the titration with Y118A (150 $\mu M$) and D121A (100 $\mu M$). The thermodynamic parameters from the fittings of the ITC data are shown in Table 2.

**Table S1. Interface analyses of VHH4 using PDBePISA.**

| VHH4  (chain B) | HSDC^a^ | ASA (Å^2^)^b^ | BSA (Å^2^)^c^ | Buried area  Percentage^d^ | ΔiG (kcal/mol)^e^ |
| --- | --- | --- | --- | --- | --- |
| B:VAL   2 |  | 78.66 | 0.00 |  | 0.00 |
| B:GLN   3 |  | 124.13 | 0.00 |  | 0.00 |
| B:LEU   4 |  | 13.07 | 0.00 |  | 0.00 |
| B:VAL   5 |  | 83.22 | 0.00 |  | 0.00 |
| B:GLU   6 |  | 20.73 | 0.00 |  | 0.00 |
| B:SER   7 |  | 59.18 | 0.00 |  | 0.00 |
| B:GLY   8 |  | 38.93 | 0.00 |  | 0.00 |
| B:GLY   9 |  | 28.22 | 0.00 |  | 0.00 |
| B:GLY  10 |  | 31.20 | 0.00 |  | 0.00 |
| B:LEU  11 |  | 123.95 | 0.00 |  | 0.00 |
| B:VAL  12 |  | 30.01 | 0.00 |  | 0.00 |
| B:GLN  13 |  | 116.18 | 0.00 |  | 0.00 |
| B:ALA  14 |  | 43.17 | 0.00 |  | 0.00 |
| B:GLY  15 |  | 63.11 | 0.00 |  | 0.00 |
| B:GLY  16 |  | 20.36 | 0.00 |  | 0.00 |
| B:SER  17 |  | 56.41 | 0.00 |  | 0.00 |
| B:LEU  18 |  | 24.41 | 0.00 |  | 0.00 |
| B:ARG  19 |  | 132.28 | 0.00 |  | 0.00 |
| B:LEU  20 |  | 0.00 | 0.00 |  | 0.00 |
| B:SER  21 |  | 19.60 | 0.00 |  | 0.00 |
| B:CYS  22 |  | 0.98 | 0.00 |  | 0.00 |
| B:ALA  23 |  | 37.25 | 0.00 |  | 0.00 |
| B:ALA  24 |  | 4.29 | 0.00 |  | 0.00 |
| B:SER  25 |  | 64.96 | 0.00 |  | 0.00 |
| B:GLY  26 |  | 56.51 | 0.00 |  | 0.00 |
| B:LEU  27 |  | 35.18 | 0.00 |  | 0.00 |
| B:THR  28 |  | 136.54 | 0.00 |  | 0.00 |
| B:VAL  29 |  | 55.92 | 0.00 |  | 0.00 |
| B:ASP  30 |  | 127.09 | 0.00 |  | 0.00 |
| B:ASP  31 |  | 29.21 | 0.00 |  | 0.00 |
| B:TYR  32 |  | 21.02 | 0.00 |  | 0.00 |
| B:ALA  33 |  | 4.85 | 4.68 | \|\|\|\|\|\|\|\|\|\| | 0.07 |
| B:ILE  34 |  | 8.68 | 0.00 |  | 0.00 |
| B:GLY  35 |  | 0.00 | 0.00 |  | 0.00 |
| B:TRP  36 |  | 0.00 | 0.00 |  | 0.00 |
| B:PHE  37 |  | 0.00 | 0.00 |  | 0.00 |
| B:ARG  38 |  | 17.09 | 0.00 |  | 0.00 |
| B:GLN  39 |  | 66.46 | 12.39 | \|\| | -0.14 |
| B:ALA  40 |  | 26.66 | 0.00 |  | 0.00 |
| B:PRO  41 |  | 127.94 | 0.00 |  | 0.00 |
| B:GLY  42 |  | 85.58 | 0.00 |  | 0.00 |
| B:LYS  43 |  | 136.89 | 0.00 |  | 0.00 |
| B:GLU  44 |  | 154.39 | 0.00 |  | 0.00 |
| B:ARG  45 |  | 67.91 | 4.81 | \| | -0.18 |
| B:GLU  46 |  | 61.21 | 0.00 |  | 0.00 |
| B:GLY  47 |  | 12.16 | 0.00 |  | 0.00 |
| B:VAL  48 |  | 0.00 | 0.00 |  | 0.00 |
| B:SER  49 |  | 0.00 | 0.00 |  | 0.00 |
| B:CYS  50 |  | 2.30 | 2.30 | \|\|\|\|\|\|\|\|\|\|\| | 0.09 |
| B:ILE  51 |  | 22.57 | 0.00 |  | 0.00 |
| B:SER  52 |  | 3.19 | 0.00 |  | 0.00 |
| B:SER  53 |  | 39.45 | 0.00 |  | 0.00 |
| B:SER  54 |  | 60.83 | 0.00 |  | 0.00 |
| B:ASN  55 |  | 66.92 | 0.00 |  | 0.00 |
| B:GLY  56 |  | 50.83 | 0.00 |  | 0.00 |
| B:SER  57 |  | 21.23 | 0.00 |  | 0.00 |
| B:THR  58 |  | 65.20 | 0.00 |  | 0.00 |
| B:TYR  59 |  | 58.82 | 0.00 |  | 0.00 |
| B:TYR  60 |  | 44.02 | 0.00 |  | 0.00 |
| B:ALA  61 |  | 5.36 | 0.00 |  | 0.00 |
| B:ASP  62 |  | 125.58 | 0.00 |  | 0.00 |
| B:SER  63 |  | 62.46 | 0.00 |  | 0.00 |
| B:VAL  64 |  | 0.00 | 0.00 |  | 0.00 |
| B:LYS  65 |  | 121.90 | 0.00 |  | 0.00 |
| B:GLY  66 |  | 84.13 | 0.00 |  | 0.00 |
| B:ARG  67 |  | 37.62 | 0.00 |  | 0.00 |
| B:PHE  68 |  | 3.19 | 0.00 |  | 0.00 |
| B:THR  69 |  | 78.12 | 0.00 |  | 0.00 |
| B:ILE  70 |  | 12.39 | 0.00 |  | 0.00 |
| B:SER  71 |  | 54.70 | 0.00 |  | 0.00 |
| B:SER  72 |  | 37.98 | 0.00 |  | 0.00 |
| B:ASP  73 |  | 48.09 | 0.00 |  | 0.00 |
| B:ASN  74 |  | 80.55 | 0.00 |  | 0.00 |
| B:ALA  75 |  | 95.63 | 0.00 |  | 0.00 |
| B:LYS  76 |  | 136.75 | 0.00 |  | 0.00 |
| B:ASN  77 |  | 79.62 | 0.00 |  | 0.00 |
| B:THR  78 |  | 17.85 | 0.00 |  | 0.00 |
| B:ALA  79 |  | 1.84 | 0.00 |  | 0.00 |
| B:TYR  80 |  | 34.75 | 0.00 |  | 0.00 |
| B:LEU  81 |  | 0.00 | 0.00 |  | 0.00 |
| B:GLN  82 |  | 61.17 | 0.00 |  | 0.00 |
| B:MET  83 |  | 0.00 | 0.00 |  | 0.00 |
| B:ASN  84 |  | 67.28 | 0.00 |  | 0.00 |
| B:SER  85 |  | 61.77 | 0.00 |  | 0.00 |
| B:LEU  86 |  | 1.96 | 0.00 |  | 0.00 |
| B:LYS  87 |  | 88.16 | 0.00 |  | 0.00 |
| B:PRO  88 |  | 54.44 | 0.00 |  | 0.00 |
| B:GLU  89 |  | 123.64 | 0.00 |  | 0.00 |
| B:ASP  90 |  | 2.33 | 0.00 |  | 0.00 |
| B:THR  91 |  | 48.05 | 0.00 |  | 0.00 |
| B:ALA  92 |  | 5.18 | 0.00 |  | 0.00 |
| B:VAL  93 |  | 37.82 | 0.00 |  | 0.00 |
| B:TYR  94 |  | 0.00 | 0.00 |  | 0.00 |
| B:TYR  95 | H | 52.54 | 33.02 | \|\|\|\|\|\|\| | 0.29 |
| B:CYS  96 |  | 0.00 | 0.00 |  | 0.00 |
| B:ALA  97 |  | 0.00 | 0.00 |  | 0.00 |
| B:ALA  98 |  | 0.00 | 0.00 |  | 0.00 |
| B:ALA  99 |  | 12.13 | 12.13 | \|\|\|\|\|\|\|\|\|\|\| | 0.19 |
| B:VAL 100 |  | 40.72 | 8.19 | \|\|\| | 0.13 |
| B:SER 101 |  | 59.40 | 50.97 | \|\|\|\|\|\|\|\|\| | -0.07 |
| B:PRO 102 |  | 100.16 | 9.82 | \| | -0.11 |
| B:ASN 103 |  | 112.96 | 17.08 | \|\| | 0.01 |
| B:LEU 104 |  | 55.30 | 54.13 | \|\|\|\|\|\|\|\|\|\| | 0.87 |
| B:GLU 105 |  | 92.05 | 3.17 | \| | -0.03 |
| B:CYS 106 |  | 15.91 | 2.16 | \|\| | 0.03 |
| B:GLY 107 |  | 24.95 | 0.00 |  | 0.00 |
| B:THR 108 | H | 70.67 | 9.64 | \|\| | -0.10 |
| B:GLY 109 |  | 19.19 | 7.50 | \|\|\|\| | 0.11 |
| B:PRO 110 |  | 19.03 | 18.41 | \|\|\|\|\|\|\|\|\|\| | 0.29 |
| B:PHE 111 |  | 15.49 | 7.05 | \|\|\|\|\| | 0.11 |
| B:GLY 112 |  | 21.15 | 0.00 |  | 0.00 |
| B:ILE 113 |  | 158.93 | 0.00 |  | 0.00 |
| B:TYR 114 |  | 147.01 | 23.36 | \|\| | 0.37 |
| B:ALA 115 |  | 25.30 | 0.00 |  | 0.00 |
| B:SER 116 |  | 102.72 | 40.68 | \|\|\|\| | -0.03 |
| B:TYR 117 | H | 182.54 | 132.28 | \|\|\|\|\|\|\|\| | 1.01 |
| B:TYR 118 | H | 115.33 | 99.45 | \|\|\|\|\|\|\|\|\| | 0.51 |
| B:GLY 119 | H | 53.08 | 48.89 | \|\|\|\|\|\|\|\|\|\| | 0.11 |
| B:MET 120 |  | 24.78 | 22.65 | \|\|\|\|\|\|\|\|\|\| | -0.04 |
| B:ASP 121 | HS | 73.09 | 44.21 | \|\|\|\|\|\|\| | -0.33 |
| B:TYR 122 |  | 84.92 | 0.00 |  | 0.00 |
| B:TRP 123 |  | 101.79 | 54.33 | \|\|\|\|\|\| | 0.54 |
| B:GLY 124 |  | 9.22 | 4.06 | \|\|\|\|\| | -0.05 |
| B:GLN 125 |  | 180.03 | 3.55 | \| | -0.01 |
| B:GLY 126 |  | 21.49 | 0.00 |  | 0.00 |
| B:THR 127 |  | 27.46 | 0.00 |  | 0.00 |
| B:GLN 128 |  | 90.32 | 0.00 |  | 0.00 |
| B:VAL 129 |  | 0.00 | 0.00 |  | 0.00 |
| B:THR 130 |  | 47.69 | 0.00 |  | 0.00 |
| B:VAL 131 |  | 6.42 | 0.00 |  | 0.00 |
| B:SER 132 |  | 45.19 | 0.00 |  | 0.00 |
| B:SER 133 |  | 126.59 | 0.00 |  | 0.00 |
| Total |  | 6886.8 | 730.9 |  | -3.7 |

1. Residues making Hydrogen bond, Disulfide bond, Salt bridge or Covalent link
2. Solvent Accessible Surface Area
3. Buried Surface Area
4. Buried Surface percentage, one bar per 10 %
5. Solvent energy effect

**Table S2. Interface analyses of CFH using PDBePISA.**

| CFH  (chain A) | HSDC^a^ | ASA (Å^2^)^b^ | BSA (Å^2^)^c^ | Buried area  Percentage^d^ | ΔiG (kcal/mol)^e^ |
| --- | --- | --- | --- | --- | --- |
| A:SER1105 |  | 168.45 | 0.00 |  | 0.00 |
| A:THR1106 |  | 99.26 | 0.00 |  | 0.00 |
| A:GLY1107 |  | 17.42 | 0.00 |  | 0.00 |
| A:LYS1108 |  | 122.67 | 0.00 |  | 0.00 |
| A:CYS1109 |  | 7.00 | 0.00 |  | 0.00 |
| A:GLY1110 |  | 40.44 | 0.00 |  | 0.00 |
| A:PRO1111 |  | 86.85 | 0.00 |  | 0.00 |
| A:PRO1112 |  | 18.69 | 0.00 |  | 0.00 |
| A:PRO1113 |  | 45.39 | 0.00 |  | 0.00 |
| A:PRO1114 |  | 127.62 | 0.00 |  | 0.00 |
| A:ILE1115 |  | 25.69 | 0.00 |  | 0.00 |
| A:ASP1116 |  | 111.69 | 0.00 |  | 0.00 |
| A:ASN1117 |  | 39.17 | 0.00 |  | 0.00 |
| A:GLY1118 |  | 16.23 | 0.00 |  | 0.00 |
| A:ASP1119 |  | 99.65 | 0.00 |  | 0.00 |
| A:ILE1120 |  | 29.67 | 0.00 |  | 0.00 |
| A:THR1121 |  | 79.53 | 0.00 |  | 0.00 |
| A:SER1122 |  | 55.28 | 0.00 |  | 0.00 |
| A:PHE1123 |  | 154.52 | 0.00 |  | 0.00 |
| A:PRO1124 |  | 91.32 | 0.00 |  | 0.00 |
| A:LEU1125 |  | 89.33 | 0.00 |  | 0.00 |
| A:SER1126 |  | 88.28 | 0.00 |  | 0.00 |
| A:VAL1127 |  | 79.76 | 0.00 |  | 0.00 |
| A:TYR1128 |  | 17.80 | 0.00 |  | 0.00 |
| A:ALA1129 |  | 52.87 | 0.00 |  | 0.00 |
| A:PRO1130 |  | 65.69 | 0.00 |  | 0.00 |
| A:ALA1131 |  | 79.48 | 0.00 |  | 0.00 |
| A:SER1132 |  | 24.98 | 0.00 |  | 0.00 |
| A:SER1133 |  | 51.58 | 0.00 |  | 0.00 |
| A:VAL1134 |  | 3.85 | 0.00 |  | 0.00 |
| A:GLU1135 |  | 65.75 | 0.00 |  | 0.00 |
| A:TYR1136 |  | 3.02 | 0.00 |  | 0.00 |
| A:GLN1137 |  | 118.72 | 0.00 |  | 0.00 |
| A:CYS1138 |  | 18.73 | 0.00 |  | 0.00 |
| A:GLN1139 |  | 96.29 | 0.00 |  | 0.00 |
| A:ASN1140 |  | 122.77 | 0.00 |  | 0.00 |
| A:LEU1141 |  | 44.21 | 0.00 |  | 0.00 |
| A:TYR1142 |  | 44.55 | 0.00 |  | 0.00 |
| A:GLN1143 |  | 89.18 | 0.00 |  | 0.00 |
| A:LEU1144 |  | 53.02 | 0.00 |  | 0.00 |
| A:GLU1145 |  | 92.26 | 0.00 |  | 0.00 |
| A:GLY1146 |  | 60.28 | 0.00 |  | 0.00 |
| A:ASN1147 |  | 100.64 | 0.00 |  | 0.00 |
| A:LYS1148 |  | 107.75 | 0.00 |  | 0.00 |
| A:ARG1149 |  | 135.62 | 0.00 |  | 0.00 |
| A:ILE1150 |  | 0.34 | 0.00 |  | 0.00 |
| A:THR1151 |  | 8.90 | 0.00 |  | 0.00 |
| A:CYS1152 |  | 0.00 | 0.00 |  | 0.00 |
| A:ARG1153 |  | 144.60 | 0.00 |  | 0.00 |
| A:ASN1154 |  | 142.37 | 0.00 |  | 0.00 |
| A:GLY1155 |  | 34.44 | 0.00 |  | 0.00 |
| A:GLN1156 |  | 134.36 | 0.00 |  | 0.00 |
| A:TRP1157 |  | 42.99 | 0.00 |  | 0.00 |
| A:SER1158 |  | 52.27 | 0.00 |  | 0.00 |
| A:GLU1159 |  | 133.30 | 0.00 |  | 0.00 |
| A:PRO1160 |  | 40.05 | 0.00 |  | 0.00 |
| A:PRO1161 |  | 9.77 | 0.00 |  | 0.00 |
| A:LYS1162 |  | 119.24 | 0.00 |  | 0.00 |
| A:CYS1163 |  | 6.58 | 0.00 |  | 0.00 |
| A:LEU1164 |  | 26.61 | 0.00 |  | 0.00 |
| A:HIS1165 |  | 74.53 | 0.00 |  | 0.00 |
| A:PRO1166 |  | 15.44 | 0.00 |  | 0.00 |
| A:CYS1167 |  | 1.67 | 0.00 |  | 0.00 |
| A:VAL1168 |  | 112.47 | 0.00 |  | 0.00 |
| A:ILE1169 |  | 17.36 | 11.58 | \|\|\|\|\|\|\| | -0.05 |
| A:SER1170 |  | 30.15 | 0.00 |  | 0.00 |
| A:ARG1171 |  | 134.79 | 67.11 | \|\|\|\|\| | 0.62 |
| A:GLU1172 |  | 130.49 | 0.00 |  | 0.00 |
| A:ILE1173 |  | 63.32 | 0.00 |  | 0.00 |
| A:MET1174 |  | 13.87 | 13.87 | \|\|\|\|\|\|\|\|\|\| | 0.22 |
| A:GLU1175 |  | 99.96 | 0.00 |  | 0.00 |
| A:ASN1176 |  | 103.65 | 0.00 |  | 0.00 |
| A:TYR1177 |  | 82.95 | 0.00 |  | 0.00 |
| A:ASN1178 |  | 39.11 | 0.00 |  | 0.00 |
| A:ILE1179 |  | 0.00 | 0.00 |  | 0.00 |
| A:ALA1180 |  | 29.22 | 28.71 | \|\|\|\|\|\|\|\|\|\| | 0.46 |
| A:LEU1181 | H | 36.09 | 36.09 | \|\|\|\|\|\|\|\|\|\|\| | 0.20 |
| A:ARG1182 | HS | 114.50 | 65.34 | \|\|\|\|\|\| | -1.22 |
| A:TRP1183 | H | 244.52 | 242.39 | \|\|\|\|\|\|\|\|\|\| | 2.56 |
| A:THR1184 |  | 48.30 | 17.93 | \|\|\|\| | 0.25 |
| A:ALA1185 |  | 112.75 | 46.52 | \|\|\|\|\| | 0.55 |
| A:LYS1186 |  | 182.13 | 0.00 |  | 0.00 |
| A:GLN1187 | H | 42.70 | 16.02 | \|\|\|\| | -0.17 |
| A:LYS1188 |  | 134.05 | 3.78 | \| | 0.06 |
| A:LEU1189 |  | 130.86 | 84.88 | \|\|\|\|\|\|\| | 1.36 |
| A:TYR1190 |  | 83.12 | 0.00 |  | 0.00 |
| A:SER1191 |  | 5.76 | 0.00 |  | 0.00 |
| A:ARG1192 |  | 83.84 | 0.00 |  | 0.00 |
| A:THR1193 |  | 24.39 | 0.00 |  | 0.00 |
| A:GLY1194 |  | 54.64 | 0.00 |  | 0.00 |
| A:GLU1195 |  | 87.49 | 0.00 |  | 0.00 |
| A:SER1196 |  | 37.00 | 0.00 |  | 0.00 |
| A:VAL1197 |  | 0.00 | 0.00 |  | 0.00 |
| A:GLU1198 |  | 51.31 | 0.00 |  | 0.00 |
| A:PHE1199 |  | 2.45 | 0.00 |  | 0.00 |
| A:VAL1200 |  | 55.76 | 42.71 | \|\|\|\|\|\|\|\| | 0.68 |
| A:CYS1201 |  | 29.96 | 10.20 | \|\|\|\| | -0.12 |
| A:LYS1202 |  | 62.86 | 7.87 | \|\| | 0.13 |
| A:ARG1203 | H | 221.35 | 137.97 | \|\|\|\|\|\|\| | -0.02 |
| A:GLY1204 |  | 23.00 | 0.73 | \| | -0.01 |
| A:TYR1205 |  | 59.02 | 0.00 |  | 0.00 |
| A:ARG1206 |  | 99.87 | 0.00 |  | 0.00 |
| A:LEU1207 |  | 64.40 | 0.00 |  | 0.00 |
| A:SER1208 |  | 21.33 | 0.00 |  | 0.00 |
| A:SER1209 |  | 106.74 | 0.00 |  | 0.00 |
| A:ARG1210 |  | 245.03 | 0.00 |  | 0.00 |
| A:SER1211 |  | 34.45 | 0.00 |  | 0.00 |
| A:HIS1212 |  | 93.62 | 0.00 |  | 0.00 |
| A:THR1213 |  | 97.06 | 0.00 |  | 0.00 |
| A:LEU1214 |  | 52.49 | 0.00 |  | 0.00 |
| A:ARG1215 |  | 149.65 | 0.00 |  | 0.00 |
| A:THR1216 |  | 10.55 | 0.00 |  | 0.00 |
| A:THR1217 |  | 72.82 | 0.00 |  | 0.00 |
| A:CYS1218 |  | 0.59 | 0.00 |  | 0.00 |
| A:TRP1219 |  | 197.37 | 0.00 |  | 0.00 |
| A:ASP1220 |  | 48.32 | 0.00 |  | 0.00 |
| A:GLY1221 |  | 39.15 | 0.00 |  | 0.00 |
| A:LYS1222 |  | 141.20 | 0.00 |  | 0.00 |
| A:LEU1223 |  | 17.84 | 0.00 |  | 0.00 |
| A:GLU1224 |  | 85.53 | 0.00 |  | 0.00 |
| A:TYR1225 |  | 34.35 | 0.00 |  | 0.00 |
| A:PRO1226 |  | 0.33 | 0.00 |  | 0.00 |
| A:THR1227 |  | 43.78 | 0.00 |  | 0.00 |
| A:CYS1228 |  | 4.42 | 0.00 |  | 0.00 |
| A:ALA1229 |  | 38.82 | 0.00 |  | 0.00 |
| A:LYS1230 |  | 133.72 | 0.00 |  | 0.00 |
| A:ARG1231 |  | 203.78 | 0.00 |  | 0.00 |
| Total |  | 8952.8 | 833.7 |  | -5.5 |

1. Residues making Hydrogen bond, Disulfide bond, Salt bridge or Covalent link
2. Solvent Accessible Surface Area
3. Buried Surface Area
4. Buried Surface percentage, one bar per 10 %
5. Solvent energy effect

Reference

1. Kelly, S. M., Jess, T. J., and Price, N. C. (2005) How to study proteins by circular dichroism. *Biochim. Biophys. Acta - Proteins Proteomics*. **1751**, 119–139

2. Yoshida, Y., Miyata, T., Matsumoto, M., Shirotani-Ikejima, H., Uchida, Y., Ohyama, Y., Kokubo, T., and Fujimura, Y. (2015) A novel quantitative hemolytic assay coupled with restriction fragment length polymorphisms analysis enabled early diagnosis of atypical hemolytic uremic syndrome and identified unique predisposing mutations in Japan. *PLoS One*. **10**, 1–21

3. Akiba, H., Tamura, H., Kiyoshi, M., Yanaka, S., Sugase, K., Caaveiro, J. M. M., and Tsumoto, K. (2019) Structural and thermodynamic basis for the recognition of the substrate-binding cleft on hen egg lysozyme by a single-domain antibody. *Sci. Rep.* **9**, 4–6
